# Long-Term Administration of Triterpenoids From *Ganoderma lucidum* Mitigates Age-Associated Brain Physiological Decline via Regulating Sphingolipid Metabolism and Enhancing Autophagy in Mice

**DOI:** 10.3389/fnagi.2021.628860

**Published:** 2021-05-06

**Authors:** Miao Zeng, Longkai Qi, Yinrui Guo, Xiangxiang Zhu, Xiaocui Tang, Tianqiao Yong, Yizhen Xie, Qingping Wu, Mei Zhang, Diling Chen

**Affiliations:** ^1^School of Pharmacy, Chengdu University of Traditional Chinese Medicine, Chengdu, China; ^2^State Key Laboratory of Applied Microbiology Southern China, Institute of Microbiology, Guangdong Academy of Sciences, Guangzhou, China; ^3^Guangdong Provincial Key Laboratory of Microbial Culture Collection and Application, Institute of Microbiology, Guangdong Academy of Sciences, Guangzhou, China; ^4^Guangdong Open Laboratory of Applied Microbiology, Institute of Microbiology, Guangdong Academy of Sciences, Guangzhou, China; ^5^School of Basic Medical Science, Guangzhou University of Chinese Medicine, Guangzhou, China; ^6^Academy of Life Sciences, Jinan University, Guangzhou, China

**Keywords:** *Ganoderma lucidum*, triterpenoids, aging, mTOR pathway, sphingolipid metabolism

## Abstract

With the advent of the aging society, how to grow old healthily has become an important issue for the whole of society. Effective intervention strategies for healthy aging are most desired, due to the complexity and diversity of genetic information, it is a pressing concern to find a single drug or treatment to improve longevity. In this study, long-term administration of triterpenoids of *Ganoderma lucidum* (TGL) can mitigate brain physiological decline in normal aging mice. In addition, the age-associated pathological features, including cataract formation, hair loss, and skin relaxation, brown adipose tissue accumulation, the β-galactosidase staining degree of kidney, the iron death of spleen, and liver functions exhibit improvement. We used the APP/PS1 mice and 3 × Tg-AD mice model of Alzheimer’s Disease (AD) to further verify the improvement of brain function by TGL and found that Ganoderic acid A might be the effective constituent of TGL for anti-aging of the brain in the 3 × Tg-AD mice. A potential mechanism of action may involve the regulation of sphingolipid metabolism, prolonging of telomere length, and enhance autophagy, which allows for the removal of pathological metabolites.

## Introduction

The physiological function of human organs gradually declines with aging, and the brain is no exception, as it shows a decline in learning ability, memory, attention, decision-making speed, sensory perception (i.e., vision, hearing, touch, smell, and taste), and motor coordination (Mattson and Arumugam, 2018). As individuals age, there is a decline in many cognitive skills, including executive function, working memory (especially task switching), and episodic memory (van Geldorp et al., 2015). It is hard for old people to understand fast language and complex sentences due to cognitive decline and hearing loss (Parker et al., 2006; Puvill et al., 2016; Maharani et al., 2018). Age-related decline in brain function occurs at about the same time as a decline in activity in other organs and then accelerates significantly after age 50 (Bowtell et al., 2017). Thus, keeping the brain in a normal physiological state is an important index used to evaluate healthy aging in terms of activities of daily living.

When individuals enter their 60, 70, and/or 80 s, physiological aging is accompanied by an increase in the potential for neurodegenerative diseases, such as Alzheimer’s disease (AD) and Parkinson’s disease (PD) (Mattson and Arumugam, 2018; Jia et al., 2020). Aging is also a major risk factor for stroke (Mancino et al., 2018; Hou et al., 2019). The proportion of individuals over age 65 in most industrialized countries is increasing rapidly, and this age group is considered to be at the ‘risk period’ for higher rates of AD, PD, and stroke (Puvill et al., 2016; Mattson and Arumugam, 2018; Jia et al., 2020). It has been estimated that over 12 million Americans will be diagnosed with AD in the next 30 years. The number of deaths caused by AD has increased by 70% in the United States between 2000 and 2013 (Strydom et al., 2018). Currently, there are about 1 million people with PD in the United States (Abeliovich and Gitler, 2016; Marras et al., 2018), and every year, about 12 million people experience strokes while nearly 3 million people die (Sarraj et al., 2020). The aging process in China is much faster than we imagined, in which more than 249 million elderly people over the age of 60, which account for 17.88% of the total population and make China the country with the largest aging population in the world (Chen et al., 2019). In 2005, the prevalence of AD was reported to be 3.5% following a large-sample, population-based survey in four regions including rural and urban areas (Zhang et al., 2005). Two large-sample, multi-region studies have been performed: the first was in 2014 and the second in 2019. Studies have shown that the prevalence of dementia was 5.14% (95% confidence interval (CI) 4.71–5.57) in 2014 and 5.60% (95% CI 3.50–7.60) in 2019 for individuals aged 65 years or older (Jia et al., 2014; Huang et al., 2019). Due to the aging of Chinese society, the current incidence of dementia may be higher (Jia et al., 2020). The latest global burden of disease study in 2019 showed that the prevalence of age-standardized dementia in China increased by 5.6% from 1990 to 2016, and the global prevalence increased by 1.7% (Afshin et al., 2019; Feigin et al., 2019; Zhou et al., 2019). Therefore, effective intervention strategies for healthy aging are most desired, especially in China.

Thanks to the developments in science and technology, many characteristics of the aging brain have been identified at the cellular, molecular, and even global organ level, including the following: (1) mitochondrial dysfunction (A. Grimm and Eckert, 2017; Mattson and Arumugam, 2018; Stockburger et al., 2018); (2) protein, nucleic acid, and lipid damage and accumulation by oxidation in cells (A. Grimm and Eckert, 2017; Tse and Herrup, 2017; Zucca et al., 2017; Mattson and Arumugam, 2018); (3) energy metabolism disorders (Yin et al., 2016; Mattson and Arumugam, 2018; Shetty et al., 2019); (4) impaired cellular “waste disposal” mechanisms (autophagy-lysosome and proteasome dysfunction) (Lipinski et al., 2010; Ling and Salvaterra, 2011; Yang et al., 2014; Mattson and Arumugam, 2018); (5) impaired adaptive stress response signaling pathways (Yin et al., 2016; Mattson and Arumugam, 2018; Lautrup et al., 2019); (6) DNA repair impairments (Maynard et al., 2015; Puvill et al., 2016; Mattson and Arumugam, 2018); (7) abnormal neural network activity (Puvill et al., 2016; Mattson and Arumugam, 2018); (8) dysregulation of neuronal calcium ion levels (van Geldorp et al., 2015; Puvill et al., 2016; Mattson and Arumugam, 2018); (9) stem cell depletion (Puvill et al., 2016; Mattson and Arumugam, 2018); and (10) increased inflammatory responses (Puvill et al., 2016; Mattson and Arumugam, 2018). Cell senescence and telomere wear are two markers of proliferative peripheral tissue senescence in humans and may occur in some types of glial cells in the brain (Conklin et al., 2018; Anitha et al., 2019), but this remains to be confirmed. Thus, any diet, exercises, medicines, interventions, and composite strategies on improving the indicators mentioned above are valuable as therapeutic interventions to be investigated.

Sun Simiao, a famous Chinese physician and considered the ancient pharmaceutical King of Chinese Medicine, sustained that for disease prevention, food intervention is to be the first approach, followed by the homology of medicine and food, and lastly drugs. Thus, the homology of medicine and food is one of the most popular choices in Asian countries. *Ganoderma lucidum* (*G. lucidum*) is a traditional Chinese medicine with a history dating back thousands of years. As medicine and food homologous resource, it has been widely used in Asian countries, such as China, Japan, and Korea, for tranquilizing and prolonging life effects (Klupp et al., 2016; Liang et al., 2019; Zeng et al., 2019). No serious toxic side effects of *G. lucidum* have been reported at present (Klupp et al., 2016; Liang et al., 2019; Zeng et al., 2019), and some studies and reviews have indicated that *G. lucidum* is safe, tolerable, and free of toxic effects (Wachtel-Galor et al., 2004; Klupp et al., 2016; Liang et al., 2019; Zeng et al., 2019; Phu et al., 2020). However, additional well-designed, large-scale randomized control trials also need to be performed to evaluate its short- and long-term pharmacological and toxicological effects.

*Ganoderma lucidum* is rich in triterpenoids (Supplementary Table 1) and exerts pharmacological activities in the heart, liver, spleen, and brain (Supplementary Tables 2, 3). In this study, the effects of long-term administration of TGL were evaluated in 8-month normal aging mice and lasting for another 10 months, observing the effect of TGL on the brain of normal aging mice. Besides, this also includes age-associated physiological indices: eye function (cataract formation), hair loss and skin relaxation, brown adipose tissue accumulation, the β-galactosidase staining degree of the kidney, and the iron death of the spleen. The potential therapeutic effects of TGL on age related-diseases were evaluated in the APP/PS1 and 3 × Tg-AD transgenic mouse model to verify the mechanism of TGL to improve brain damage.

## Materials and Methods

### TGL Preparation

Triterpenoids of *Ganoderma lucidum* preparation was carried out as follows [refer to Supplementary Figure 1A for the extraction process; the *Ganoderma* triterpene (ganoderenic acids B, C, and D and ganoderic acids A, B, C2, D, G, and H) content of each compound is shown in Supplementary Figure 1B and Supplementary Table 1] (Lai et al., 2019; Liu et al., 2021): we crushed the common feed for mice, mixed the 1 Kg feed with 2 g TGL extract evenly, added a small amount of pure water to dilute and rub, cut this into small pieces, and dried them at 60°C, and then the administered TGL feed was ready.

### Animals and Treatments

#### Normal Aging

C57 BL/6 mice (aged 25 weeks) were obtained from the Center of Laboratory Animal of Guangdong Province (SCXK [Yue] 2008-0020, SYXK [Yue] 2008-0085) and were housed in plastic cages in a temperature-controlled (25 ± 2°C) colony room, exposed to a 12/12-h light/dark cycle. Food and water were available *ad libitum*. All experimental protocols were approved by the Center of Laboratory Animals of the Guangdong Institute of Microbiology.

**FIGURE 1:**
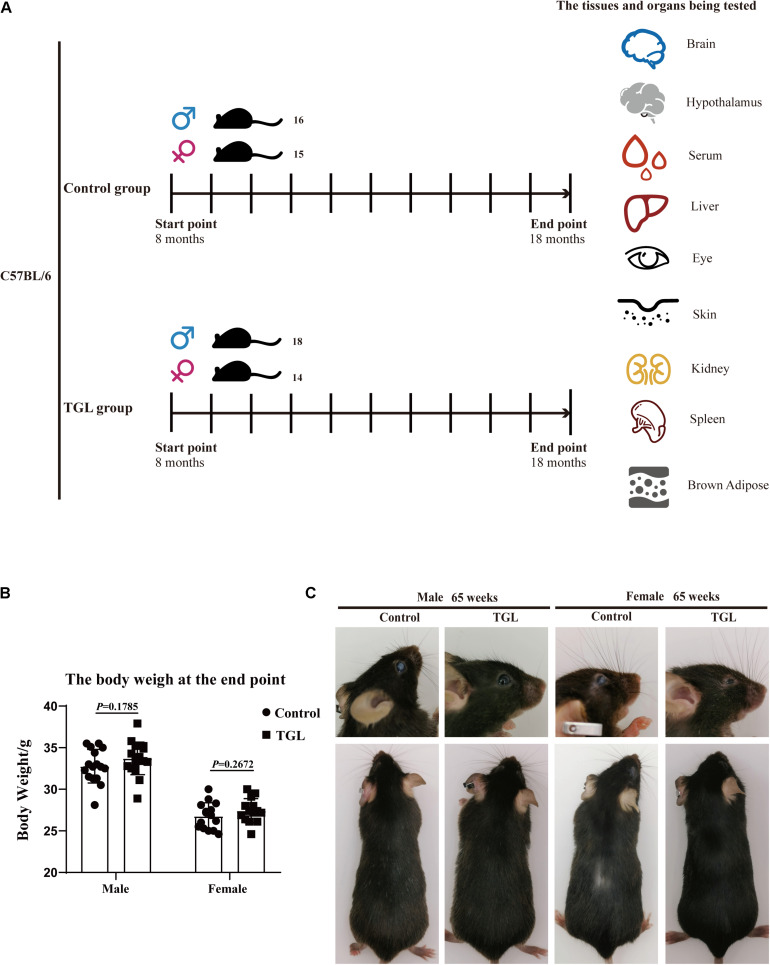
Graphical abstract and Experimental procedure in normal aged mice. **(A)** Experimental procedure in normal aged mice; **(B)** TGL shows no significant effects on the bodyweight of normal aged mice; **(C)** TGL shows an improvement in eyes and hair in normal aged mice. *n* ≥ 14.

We divided 100 mice into four groups evenly: Female control group, Female TGL group, Male control group, and Male TGL group. After 8 months of normal feeding, we began the administration of TGL. The mice of the control group were fed with a standard diet, and the mice of the TGL groups were fed a diet containing triterpenes of *G. lucidum*. Water was available *ad libitum*. These treatments lasted 10 months, some aging mice died in a 10-month experiment, thus the final number of mice and the specific grouping strategy depends on Figure 1A. The younger group (12 weeks, acclimate for at least 1 week) received no treatment and was used only for comparison in metabolomics analysis.

Blood was collected through the venous sinus of the eye-orbit after the animals were anesthetized. The blood was left at room temperature for 2 h, then centrifuged (3500 rpm, 10 min, 4°C), collected serum, equalized, and stored at −80°C. After blood sample collection, lacrimal gland, cornea, skin, brown adipose, spleen, and some brain tissue were removed, fixed in neutral formalin, dehydrated, and cleared. The samples were then mounted in paraffin and cut into sections for histological assessment; Kidney and liver tissue were stored at −80°C to prepare frozen sections; Besides 3 per group hypothalamus were placed in RNA protective solution; The remaining brain tissues were stored at −80°C after liquid nitrogen treatment.

#### APP/PS1 Double Transgenic Mouse Preparation and Treatment

Thirty male APP/PS1 transgenic mice (2 months of age) were purchased from Beijing HFK Bioscience Co., LTD. The mean bodyweight of the mice was 20 ± 5 g. Animals were allowed to acclimate for at least 4 weeks before the initiation of the experiment. APP/PS1 transgenic mice were randomly allocated into three groups of 10: AD model (Model, M), low-dose group (oral TGL at 25 mg/[kg/d]) (TGLL), and the high-dose group (oral TGL at 100 mg/[kg/d]) (TGLH). Then ten C57BL/6J male mice (9 months of age) purchased from Beijing HFK Bioscience Co., LTD., was utilized as the control group (Normal, N). The Control group and Model group were treated with equal volumes of distilled water. TGL was suspended in distilled water, and i.g once a day for 24 weeks. The steps for blood collection and hypothalamus removal are the same as above.

#### 3 × Tg-AD Mouse Preparation and Treatment

A total of 16 male 3 × Tg-AD mice (129-Tg (APPSwe, tauP301L)1Lfa Psen1tm1Mpm/Mmjax, 6 weeks of age) were purchased from Beijing HFK Bioscience Co., LTD. The mean bodyweight of the mice was 20 ± 5 g. Mice were randomly allocated into two groups of eight: Control group (Control), GA group (GA). A 10 mg/kg/d dose of ganodenic acid A, which was suspended in distilled water was administered to the GA group. The control group was treated with equal volumes of distilled water, and this lasted for 12 weeks. The steps for blood collection are the same as above, and brain tissues were stored at −80°C after liquid nitrogen treatment.

### Histopathology

Paraffin sections were gradually dewaxed to water. We strictly followed the procedures or instructions of HE staining, the Prussian blue iron staining Kit, and the Tunel Cell Apoptosis Detection Kit (which required repair in EDTA or sodium citrate solution at high temperature if the formalin soaking time is too long). Oil red O staining and β-galactosidase staining used frozen tissue sections. See the kit instructions on the Key resources table. Observe with the microscope and capture images, the images were analyzed by using ImageJ software (NIH).

### Western Blot Analysis

We referred to the protocol described on the Thermo Fisher website^1^ or Affinity Biosciences website^2^. Global brain tissue was dissected from normal aging mice and 3 × Tg-AD mice, and proteins were extracted with radioimmunoprecipitation assay (RIPA) lysis buffer (Thermo Scientific^TM^ T-PERTM Tissue Protein Extraction Reagent, 78510). The proteins were separated by sodium dodecyl sulfate-polyacrylamide gel electrophoresis and transferred onto polyvinylidene fluoride membranes followed by incubation with a horseradish peroxidase-conjugated goat anti-mouse or goat anti-rabbit IgG secondary antibody (1:10000). The antibodies can be seen in the key resources table. Band intensity was quantified using ImageJ software.

### Transcriptome Sequencing

We took the hypothalamus tissues of normal aging mice and APP/PS1 mice; total RNA was extracted with a TRIzol reagent kit (Invitrogen, Carlsbad, CA, United States). RNA concentration and purity were measured using NanoDrop 2000 (Thermo Fisher Scientific, Wilmington, DE, United States). RNA integrity was assessed using the RNA Nano 6000 Assay Kit of the Agilent Bioanalyzer 2100 system (Agilent Technologies, Santa Clara, CA, United States). A total amount of 1 μg RNA per sample was used as input material for the RNA sample preparations. Sequencing libraries were generated using NEBNext Ultra^TM^ RNA Library Prep Kit for Illumina (NEB, United States) following manufacturer’s recommendations, and index codes were added to attribute sequences to each sample. The clustering of the index-coded samples was performed on a cBot cluster generation system using TruSeq PE Cluster Kit v4-cBot-HS (Illumina) according to the manufacturer’s instructions. After cluster generation, the library preparations were sequenced on an Illumina platform and paired-end reads were generated. KEGG pathway enrichment analysis use KEGG database^3^.

### Unsupervised Metabolomics Analysis

#### Sample Preparation

Volumes of 120 μL frozen serum aliquots were mixed with 480 μL extraction solution by mixing methanol and acetonitrile (2:1). The extraction solution contained two standards (2-chloro-L-phenylalanine and decanoic acid). Samples were vortexed for 120 s and then placed at 4°C for 30 min. Following centrifugation for 10 min at 14000 rpm, the supernatant was split into two aliquots (250 μL each aliquot), and one of the supernatant aliquots was used for analysis and one for backup. The aliquot to be analyzed was dried via evaporation in Labconco Centrivap Console and was then dissolved in 125 μL 50% methanol and centrifuged at 14000 rpm for 10 min. The supernatant was moved to 200 μL MicroSert Insert for analysis.

Brain tissue (100 mg) was homogenized in 1000 μL extraction solution by mixing methanol acetonitrile (2:1), then placed at 4°C for 30 min. The extraction solution contained two standards (2-chloro-L-phenylalanine and decanoic acid). Following centrifugation for 10 min at 14000 rpm, the supernatant was split into two aliquots (400 μL each aliquot), one of the supernatant aliquots was used for analysis and the other was stored for backup. The aliquot used for analysis was dried via evaporation in Labconco Centrivap Console and then was dissolved in 200 μL 50% methanol and centrifuged at 14000 rpm for 10 min. The supernatant was moved to 200 μL MicroSert Insert for analysis.

#### Mass Spectroscopy Analysis

Sample extraction and reconstitution were performed in solvents compatible with positive and negative ionization modes and all modes were run on a Thermo UltiMate 3000 RSLC and a Thermo Scientific Q-Exactive Focus high-resolution mass spectrometer. The heated electrospray ion source of mass spectroscopy (MS) was maintained at 300°C. The capillary temperature was maintained at 320°C for both positive and negative injections. The spray voltage was 3.5 KV for positive injections and 3.2 KV for negative injections. The flow rates of sheath gas and auxiliary gas for both positive and negative injections were 45 (arbitrary units) and 8 (arbitrary units), respectively. The scan model was Full MS/dd-MS2. The mass resolution of full-MS and dd-MS2 for both positive and negative injections were set to 35,000 and 17,500, respectively. MS/MS normalized collision energy was set to 20, 40, and 60 eV. The instrument scanned 70–1050 m/z. Both positive and negative ion modes were operated with a 2.7 μm particle using a 2.1 mm × 100 mm Waters CORTECS T3 column. The column temperature was maintained at 40°C. In the positive ion mode, the sample extracts were gradient-eluted at 400 μL/min using (A) 0.1% formic acid in water and (B) 0.1% formic acid in acetonitrile (2% B-25% B in 3.5 min, 25% B-100% B in 15 min, 100% B for 3 min). In the negative ion mode, the sample extracts were gradient-eluted at 400 μL/min using (A) water and (B) acetonitrile (2% B-25% B in 3.5 min, 25% B-100% B in 15 min, 100% B for 3 min).

### Statistical Analysis

All data are described as the means ± standard deviations (SD) of at least three independent experiments. The significant differences between treatments were analyzed using one-way analysis of variance (ANOVA) or *T*-Test at *p* < 0.05. The Statistical Package for the Social Sciences (SPSS, Abacus Concepts, Berkeley, CA, United States) and Prism 8 (GraphPad, San Diego, CA, United States) software were used for all statistical analyses. The levels of significance were set at ^∗^*p* < 0.05, ^∗∗^*p* < 0.01, and ^∗∗∗^*p* < 0.001, ns means no significance.

## Results

### Weight and Appearance Changes

**FIGURE 2:**
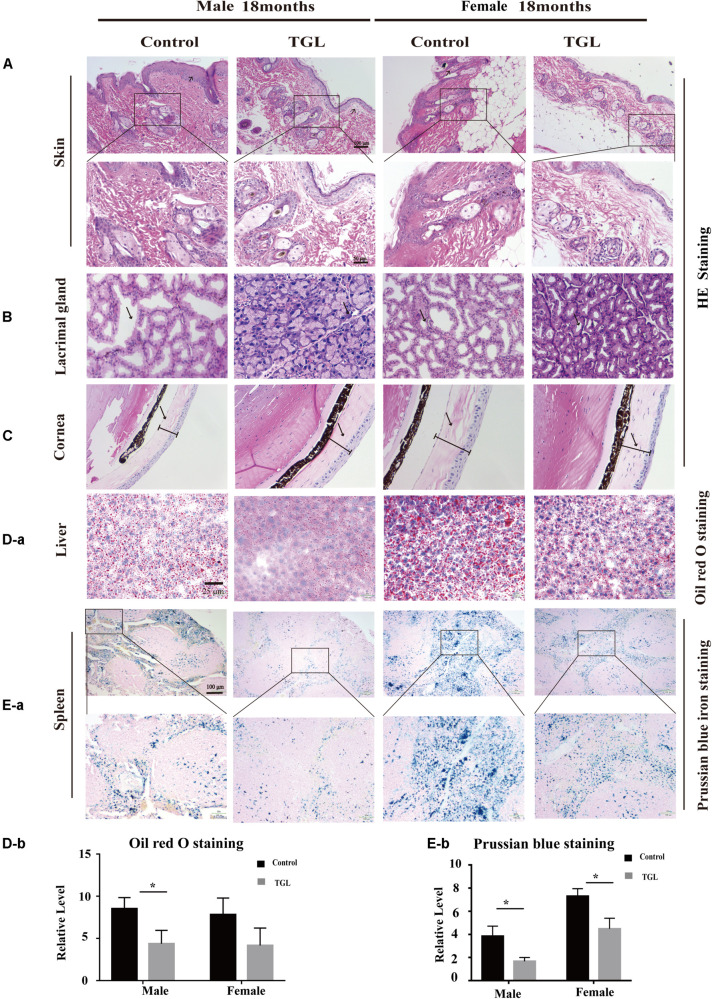
Improvement of multiple organ aging after long-term administration of TGL. TGL shows an improvement in skin, eyes, liver, and spleen. **(A)** HE staining of the skin; **(B)** HE staining of the Lacrimal gland; **(C)** HE staining of the Cornea; **(D)** Oil red O staining of the liver; **(E)** Prussian blue iron staining of the spleen. Data are presented as the means ± SD of more than six independent experiments. *p < 0.05.

In this study, we found there were no significant differences in weight between the TGL-treated group and the control group, regardless of sex (Figure 1B), but the female TGL-treated group had a slender body shape. The condition of the skin is an important indicator of aging (Bonte et al., 2019), we found that the TGL-treated group has smoother fur after 10 months of TGL administration, the fur of the control group was more dry and sparse, females responded more robustly than males in terms of appearance in the gloss of the hair, and part of the control group showed shedding of senescent cells and skin diseases (Figure 1C), hints that TGL seems to have a more pronounced effect on female aging mice. H&E staining indicated that there exist thickening and distortion of the elastic fibers and the epidermal cell layer was arranged irregularly in the control group, while in the TGL-treated groups, these symptoms showed significant improvement (Figure 2A). Besides, some mice in the control group had cataracts, while in the TGL-treated groups no cataracts were found in either males or females (Figure 1C), cataracts occurred in 11.1% of males and 14.28% of females in the control groups. Hematoxylin and eosin (H&E) staining of lacrimal glands of the control group showed obvious acinar cavity expansion and flat epithelium, while TGL treatment significantly improved the function of lacrimal glands (Figure 2B). Besides, the corneal epithelium thickened and was arranged irregularly, and the collagenous fiber arrangement of the corneal stroma lost its original shape in the Control group (Figure 2C).

### Enhanced Autophagy Effects of Triterpenoids of *G. lucidum* on the Brain of Normal Aged Mice

**FIGURE 3:**
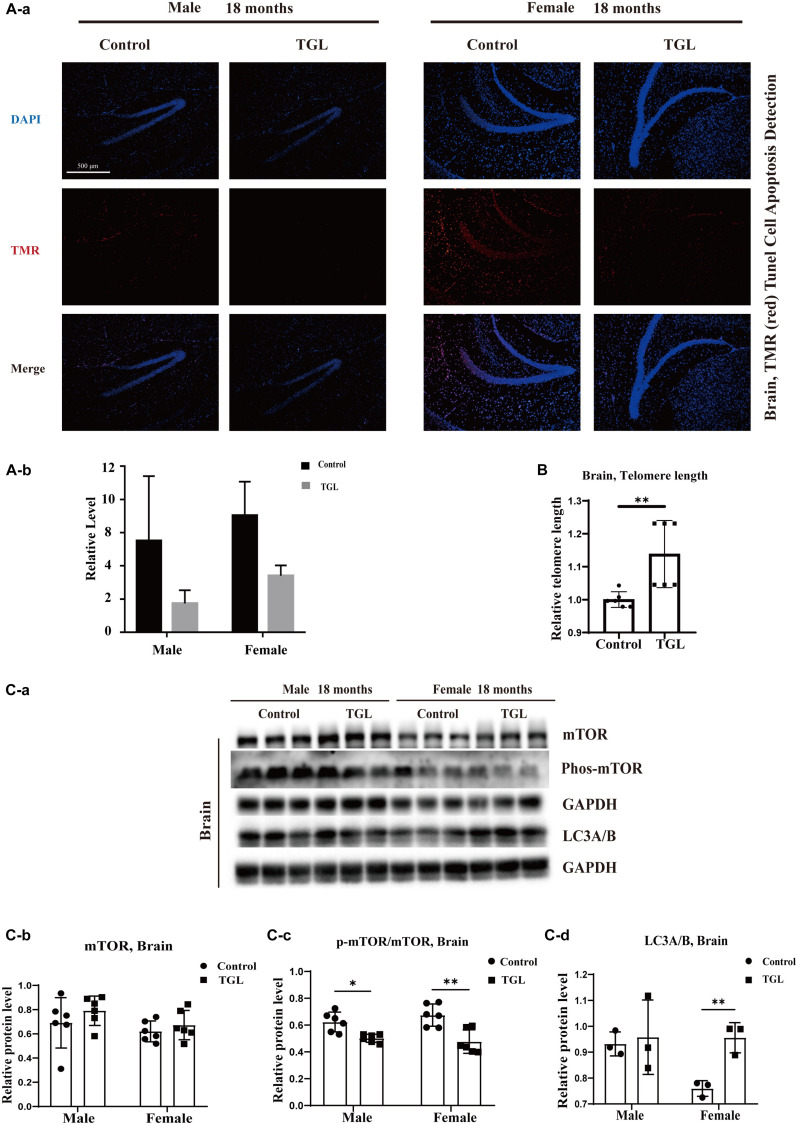
Effects of triterpenoids of *G. lucidum* on the brain of normal aged mice. **(A)** TGL shows an improvement in brain cells apoptosis rate using TUNEL staining; **(B)** TGL shows inhibition of telomere shortening in the brain; **(C)** TGL shows an impact on the mTOR pathway to enhance the autophagy. Data are presented as the means ± SD of more than six independent experiments, and more than three independent experiments in Western blot. *p < 0.05 and **p < 0.01.

A mind-tranquilizing effect was the main function of *G. lucidum* in its traditional application, and in our previous study, we showed that alcohol extracts of *G. lucidum* could delay the progression of AD by regulating DNA methylation in rodents (Lai et al., 2019). Thus, we evaluated other potential effects and mechanisms of the aging brain. Tunnel assays for the detection of apoptotic cells showed there were fewer apoptotic cells in brain tissue samples in the TGL-treated groups than that in the control group (Figure 3A, *p* < 0.05). The telomere lengths in brain tissue samples were longer in the female TGL-treated group than that in the female control group (Figure 3B, *p* < 0.05). Then we found the expression of phosphorylated-mTOR and LC3A/B were upregulated in the TGL-treated groups (Figure 3C, *p* < 0.05). Indicate that triterpenoids of *G. lucidum* may delay brain aging by delaying telomere shortening, activating autophagy, and other functions.

### Effect of Triterpenoids of *G. lucidum* on the Hypothalamus of Normal Aged Mice

**FIGURE 4:**
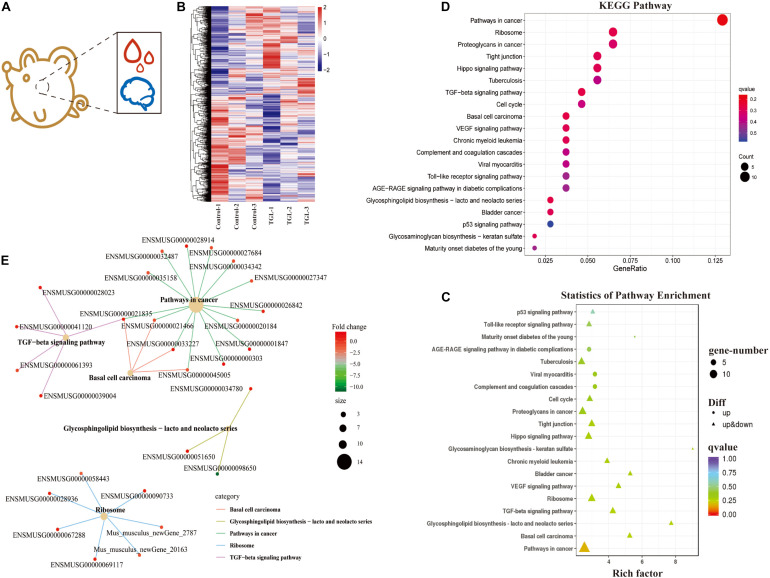
Effect of Triterpenoids of *G. lucidum* on hypothalamus RNA sequencing of normal aged mice. **(A)** Schematic diagram of test index; **(B)** Heatmap of differently expressed mRNAs; **(C,D)** Enrichment analysis of differentially expressed gene KEGG pathway. Note: The *x*-coordinate is GeneNum, indicating the number of genes of interest in this entry, and the *Y*-coordinate is each Pathway entry. The color of the column represents the *p*-value of the hypergeometric test. **(E)** KEGG enrichment network diagram of differentially expressed genes. The network diagram of differentially expressed genes and the KEGG pathway. The color of edges represents different pathways, and the color of gene nodes represents multiple differences. The larger the pathway node, the more genes enriched in the pathway.

In our previous study, we demonstrated that alcohol extracts of the *G. lucidum* fruit body could improve the microbiome-gut-liver axis, improve the microbiome-gut-brain axis, and could regulate the CNS, and improve metabolic regulation in high sugar and fat diet mice (Diling et al., 2020). Thus, we explored the mechanism of TGL involvement in metabolic regulation using RNA sequencing of extracts from hypothalamus tissue samples (Figure 4A). As shown in Figure 4B, there were about 366 differentially expressed mRNAs detected, among which 190 mRNAs were up-regulated and 176 mRNAs were down-regulated (Figure 4B and Table 1). Enrichment analysis of differentially expressed genes using KEGG (Kyoto Encyclopedia of Genes and Genomes) pathway analysis is shown in Figures 4C,D. The main pathways involved included pathways in cancer, the TGF-beta signaling pathway, and biosynthesis of the glycosphingolipid Lacto/neolacto series. The network interaction of DEGs is shown in Figure 4E. Previous studies have revealed that sphingolipid metabolism could be involved in galactosylceramide and glycosphingolipid biosynthesis, and TGL may thus target sphingolipid metabolism.

**TABLE 1:** RNA sequencing of hypothalamus (Top 100 different expressed mRNAs).

Symbol	C1_FPKM	C2_FPKM	C3_FPKM	TGL-1_FPKM	TGL-2_FPKM	TGL-3_FPKM	*p*-value	log2FC	Regulated
Mus_musculus_newGene_12923	3.889608	3.290139	3.234409	0.053785	0	0.056914	1.66E-73	−6.57187	down
Gm43720	0.669973	0.584832	0.734276	0.02567	0	0.000223	3.09E-32	−5.97605	down
Taok1	11.70314	12.37501	10.5112	2.730262	3.997773	3.198907	1.02E-26	−1.80556	down
Gm28048	8.712056	9.694984	0.431736	0	0.000306	0	9.32E-20	−10.7704	down
Bcl2l2	39.69579	38.87336	37.57313	17.4676	15.38983	18.45921	1.03E-19	−1.31449	down
Mus_musculus_newGene_14522	0.24126	0.611931	0.31302	0.059981	0.021117	0.060201	9.12E-11	−3.02239	down
Mus_musculus_newGene_1511	15.50723	36.33932	11.57809	5.083674	5.985105	3.695479	1.97E-10	−2.09377	down
Ngp	0.969112	0.835183	1.236629	0.114383	0.183466	0.145172	7.08E-10	−2.74507	down
Phc3	10.24863	6.610594	8.556628	2.537218	4.992046	3.632396	2.53E-09	−2.21105	down
Cpne1	4.453808	4.480745	5.728442	11.16032	7.617339	11.39168	8.88E-09	1.320762	up
Mus_musculus_newGene_15486	2.116321	1.838757	1.799755	0.587295	0.122823	0.275683	1.03E-08	−2.551	down
Anp32e	18.27329	21.48529	21.3954	34.10668	30.15924	32.04015	2.00E-08	0.846808	up
Lypd6	6.339828	2.896344	8.136196	1.943519	2.299223	2.859215	2.15E-08	−1.8172	down
Ptch1	18.11132	12.92783	19.11014	8.431311	10.36519	7.254182	3.49E-08	−1.03398	down
Gatad2b	8.531223	3.290149	7.480017	1.829689	2.707072	2.368215	1.57E-07	−1.62155	down
Mus_musculus_newGene_7451	0.47321	0.715459	0.900427	2.064423	2.864607	2.115137	4.46E-07	1.732702	up
Rnf13	34.83294	27.26737	21.82686	62.65552	44.34845	84.607	4.99E-07	1.316054	up
Mus_musculus_newGene_13835	81.60193	110.9614	135.5357	301.9333	246.8458	182.6184	6.97E-07	1.12706	up
Adam1a	11.58916	10.89115	6.950538	5.682081	5.252263	5.009995	7.52E-07	−1.05363	down
Nfat5	4.938496	6.347	3.062845	5.670891	7.865202	7.090772	1.24E-06	0.997967	up
Pcdhga8	4.389451	2.866374	6.223647	2.032655	1.719958	2.082496	2.49E-06	−1.20561	down
Aldh1a2	1.697769	1.582407	1.882824	3.327981	3.696433	7.531053	2.77E-06	1.494426	up
Osbpl6	10.07962	9.967105	8.61995	7.226177	7.813877	6.724273	3.45E-06	−0.71505	down
Mus_musculus_newGene_17313	5.096818	4.661171	7.446638	2.252344	1.991808	2.109914	3.55E-06	−1.30573	down
Rac1	15.45162	15.58981	16.55221	16.1087	68.20964	69.8126	4.17E-06	1.700827	up
Ptgds	329.8186	344.9323	408.1165	571.3699	600.5531	1385.647	4.87E-06	1.211963	up
Slc13a4	0.532723	0.965426	0.480298	1.330932	1.848271	4.333051	7.00E-06	1.923232	up
Azi2	14.78044	16.10542	16.87702	21.2957	20.55793	19.16221	7.32E-06	0.700732	up
Rab27a	6.94201	8.103768	8.159093	3.867649	5.128242	3.996698	8.69E-06	−0.83788	down
Slc22a6	0.244441	0.273731	0.12178	0.411466	0.796756	2.216438	1.08E-05	2.419438	up
Myl12a	8.356751	8.685487	9.705156	15.42321	14.62305	19.54725	1.15E-05	0.804032	up
Tmem9b	25.18691	27.73696	28.4351	32.74496	62.38393	67.81904	1.21E-05	1.014054	up
Pitx2	13.58033	12.01773	7.359167	18.30537	21.04272	22.4479	1.23E-05	0.900374	up
Spp1	4.563857	5.579025	4.672519	7.785845	9.797311	15.03454	1.29E-05	1.13448	up
Paqr7	41.38397	22.08821	48.21004	18.52836	18.22971	16.8107	1.33E-05	−0.90075	down
Pcdhgb5	2.167199	1.730722	21.07459	0.801004	1.31047	0.959021	1.51E-05	−3.01305	down
Fmod	1.767021	1.258097	1.14861	2.087399	3.682632	5.977344	1.57E-05	1.502393	up
Rhof	18.47653	12.44221	13.31581	11.19265	8.337795	8.465769	1.61E-05	−0.70898	down
Pcdhgc4	13.13775	7.538917	65.98213	5.727133	6.518003	5.458537	1.75E-05	−1.69952	down
Igfbp2	6.671881	5.23182	4.62881	9.806509	9.538176	18.76624	1.88E-05	1.204219	up
Ogn	1.283395	1.324278	0.958192	1.870447	3.054949	5.543355	2.08E-05	1.551819	up
Tfdp2	5.926766	4.901073	3.93437	1.605638	3.78065	4.35234	2.28E-05	−1.30098	down
Serping1	3.146913	3.479642	3.593491	6.167186	6.26718	6.881413	2.43E-05	0.913624	up
Atp8a1	37.30142	43.92654	15.52566	13.62472	16.1126	13.84528	2.45E-05	−1.16087	down
Gpc2	0.125598	0.25074	0.30578	0.612154	0.624173	1.115076	2.74E-05	1.756986	up
Grm2	7.521406	6.845249	4.812198	12.11454	11.00831	9.703672	2.76E-05	0.783435	up
Sco2	5.863888	6.247402	0.984866	1.153106	0.606637	0.815138	2.89E-05	−2.22673	down
Pcdhgc5	5.084746	4.174581	9.834584	3.219718	3.519621	2.560906	5.76E-05	−1.09671	down
Mus_musculus_newGene_20163	19.93332	22.75848	17.98565	11.65688	9.074512	9.381307	5.87E-05	−1.00875	down
Trim2	47.02116	41.57854	12.90792	11.70039	14.17824	12.64701	6.02E-05	−1.20445	down
Clec3b	1.548666	1.612173	2.81814	5.829777	4.277997	4.631124	6.96E-05	1.288496	up
Fxyd3	0.885017	0.274696	0.365057	1.192751	1.504817	2.304581	7.73E-05	1.694682	up
Slc6a13	1.984694	1.538029	1.57059	2.857289	3.160465	6.928728	7.80E-05	1.346279	up
Atxn7l3	12.59657	11.37764	10.24654	32.68215	11.73012	26.14544	8.59E-05	1.080079	up
H3f3b	44.60186	46.38337	51.33964	126.2611	105.5351	48.37488	9.20E-05	0.973486	up
Rprm	41.61818	37.74937	33.05315	64.0092	55.17521	51.14713	9.35E-05	0.598208	up
Synj1	50.21541	57.96886	66.0465	37.84828	45.0888	31.57636	0.000101	−0.66404	down
H2-Q1	0.267972	0.243174	0.096663	0.530357	0.631109	0.99056	0.000101	1.732081	up
Rps28	68.00393	47.808	80.13228	127.8886	92.81004	118.9684	0.000103	0.79234	up
Ppp1r9a	22.42754	28.30584	24.18876	14.83328	12.26083	5.461852	0.00011	−1.30678	down
Mus_musculus_newGene_14521	1.072404	1.001249	0.873793	0.245256	0.512898	0.27324	0.000112	−1.47976	down
Foxb1	8.975046	8.292566	5.090877	15.56052	12.96705	11.33847	0.000122	0.831645	up
Prg4	0.147244	0.419417	0.344384	0.436722	1.142215	1.880561	0.000124	1.91222	up
Mus_musculus_newGene_21979	11.31698	7.821126	8.214593	5.56862	5.952519	5.044618	0.000134	−0.69488	down
Galnt16	25.79815	34.77434	28.3016	70.09834	30.49534	63.24136	0.000135	0.943713	up
Sphk1	0.273167	0.304012	0.224675	0.893284	0.770787	0.883149	0.000149	1.531377	up
Wdr77	17.27344	8.842787	16.67199	7.451219	8.619811	7.844271	0.000152	−0.88545	down
Fsd1l	3.294207	2.83381	2.810097	3.65967	3.366155	2.839395	0.000153	1.390214	up
Gm28036	9.417948	4.500991	1.961159	2.505359	1.595868	0.785525	0.000174	−1.92552	down
Myh4	0.208298	0.222836	0.151057	0.376344	0.54702	0.467163	0.0002	1.244447	up
Slc6a12	0.264733	0.227287	0.104826	0.448564	0.611936	1.848077	0.000254	1.896547	up
Aspg	1.854029	1.874206	1.906641	3.251615	2.427306	4.529861	0.000278	0.924055	up
Tmem267	11.66432	9.798137	9.689703	13.23979	15.95576	12.43407	0.000332	0.789803	up
Fbln1	3.253842	3.430193	3.274527	4.951525	5.160303	6.023687	0.000332	0.71697	up
Ahcyl2	42.43559	15.24158	37.83194	15.79211	16.5671	17.91718	0.000343	−1.00208	down
Slc47a1	0.559734	0.964785	0.483391	1.174253	1.650622	2.769661	0.000356	1.342388	up
Nbl1	11.12559	13.55878	14.09049	18.49148	19.99199	19.77541	0.000401	0.587494	up
Mus_musculus_newGene_21883	4.859227	8.03323	3.822363	1.438963	2.150494	2.388066	0.000427	−1.34507	down
Adora1	11.45976	18.19518	11.52225	18.63551	24.41292	24.19367	0.00044	0.615842	up
Bmp6	1.181217	1.537264	1.419002	2.106625	2.299605	3.440335	0.00048	0.924294	up
Gabpb2	4.133897	3.729611	3.128166	2.178008	1.648487	2.656451	0.000485	−0.74112	down
Mdfic	0.375961	0.644931	0.561184	0.803601	1.025879	0.863842	0.000498	1.100109	up
Dyrk1a	7.841053	9.030653	8.404052	12.67455	7.112959	14.23365	0.000499	0.808919	up
Mindy3	13.38096	14.45982	14.52083	9.082119	10.74618	12.46219	0.000504	−0.61554	down
Gal3st4	1.207063	1.027611	1.531161	3.433771	1.888287	2.814491	0.000541	0.905185	up
Mus_musculus_newGene_15009	2.9457	2.854937	2.396335	1.353186	1.805244	2.001115	0.000559	−0.67321	down
Col25a1	9.488102	13.3002	9.942588	5.493827	8.633615	6.928233	0.000633	−0.68212	down
Fgfr1op	6.256988	5.914114	2.999508	2.878672	2.939719	2.694882	0.000636	−0.97513	down
Macrod2	12.08988	9.510841	4.441681	4.298766	6.074126	7.414225	0.000716	−1.18296	down
Aebp1	3.095477	4.318746	3.966831	4.587501	7.083368	6.949616	0.000748	0.733223	up
Rprd1b	10.78543	3.070225	3.991385	2.486718	2.939771	2.373803	0.000752	−1.26824	down
Eya2	0.684411	0.734553	0.746447	1.221404	1.403488	1.807	0.000796	1.031067	up
Sumo2	49.4363	25.06764	10.82903	11.17719	8.213712	15.44789	0.000802	−1.32669	down
Pcdhgb1	1.822618	3.238295	1.178243	8.429773	3.888132	2.899032	0.000838	1.277849	up
Cap1	56.2933	33.90784	30.66407	61.58003	65.04071	62.07548	0.00087	0.667503	up
Fam131b	3.792873	4.024515	5.314095	6.649393	6.251654	7.349866	0.000906	0.624582	up
Gstm2	1.168082	1.128437	1.425652	2.788445	2.056896	3.038896	0.000912	1.068272	up
Bbx	4.801812	5.454796	4.683867	3.618744	3.809623	3.062787	0.000914	−0.85461	down
Ankfn1	3.826081	5.374508	3.561852	2.733567	2.333025	1.04414	0.000936	−1.00005	down
AL732309.1	0.205796	0.447086	0.47556	1.433138	0.179026	5.598956	0.000994	2.662847	up

Gene Ontology (GO) analysis showed that the cellular components, including the extracellular exosome, the integral component of plasma membrane, Golgi apparatus, the perinuclear region of cytoplasm, protein complex, and proteinaceous extracellular matrix, were significantly influenced (Supplementary Figure 2A). The biological processes of negative regulation of transcription from RNA polymerase II promoter, homophilic cell adhesion via plasma membrane adhesion molecules, cell-cell signaling, synapse organization, cellular response to oxidative stress, neurotransmitter transport, insulin secretion, and regulation of insulin-like growth factor receptor signaling pathway were influenced (Supplementary Figure 2B). Besides, the molecular functions of calcium ion binding, protein homodimerization activity, heparin-binding, ligase activity, calmodulin-binding, histone binding, neurotransmitter: sodium symporter activity, anion: anion antiporter activity, calcium channel regulator activity, and galactosylceramide sulfotransferase activity were influenced (Supplementary Figure 2C).

### Long-Term Intake of TGL Delays the Age-Related Physiological Changes in Various Organs

**FIGURE 5:**
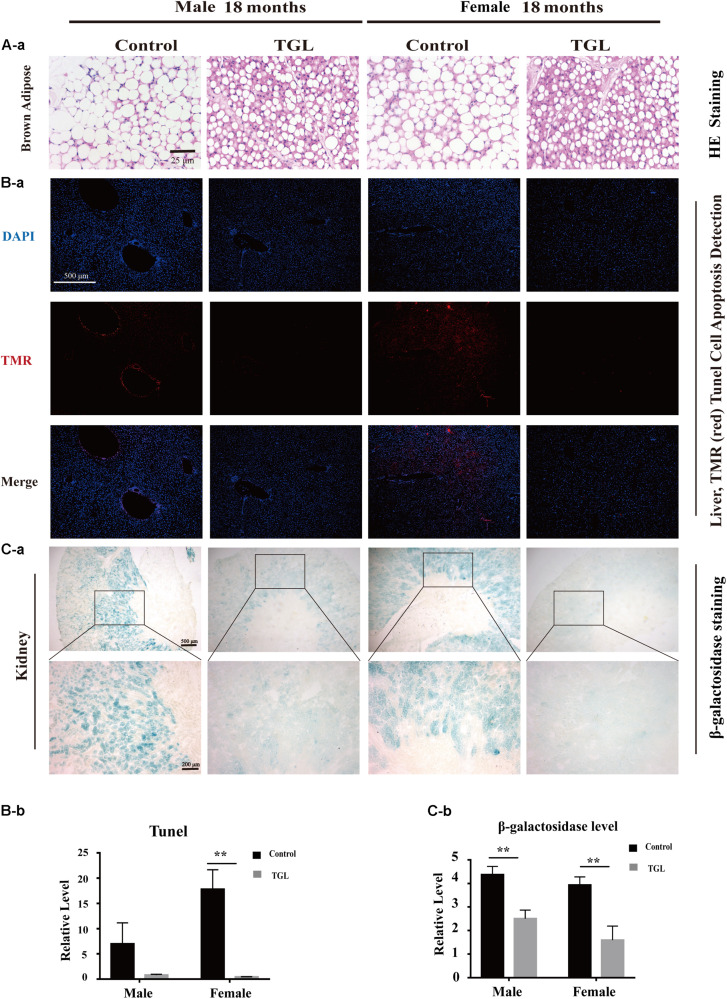
Improvement of multiple organ aging after long-term administration of TGL. TGL shows an improvement in brown adipose, liver, and kidney. **(A)** HE staining of brown adipose tissue; **(B)** Apoptosis rate of Liver using TUNEL staining; **(C)** TGL shows an improvement on aging in kidney using β-galactosidase staining. Data are presented as the means ± SD of more than six independent experiments. **p < 0.01.

**FIGURE 6:**
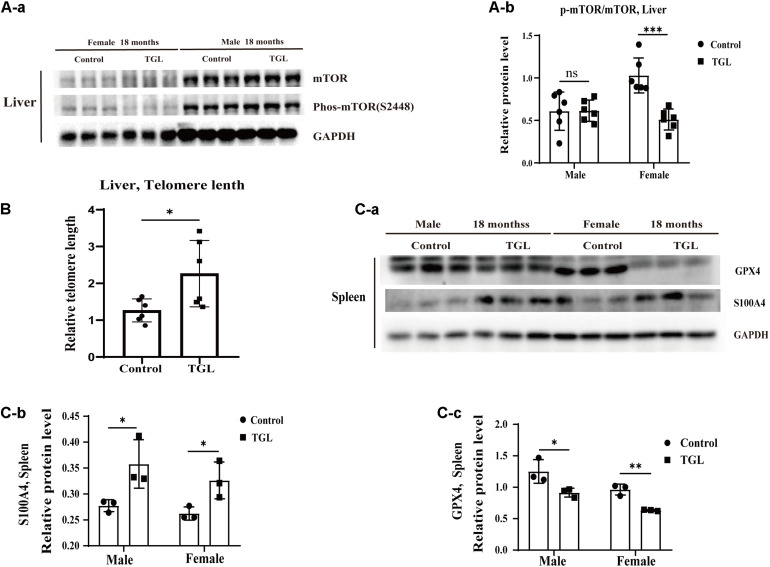
Improvement of multiple organ aging after long-term administration of TGL. TGL shows an improvement in the liver and spleen. **(A)** TGL shows inhibition of the expressions in mTOR and p-mTOR of the liver. **(B)** The expression of GPX4 and S100A4. **(C)** TGL shows inhibition of telomere shortening in the liver. Data are presented as the means ± SD of more than six independent experiments and more than three independent experiments in Western blot. *p < 0.05, **p < 0.01, ***p < 0.001, and *ns*, not statistically significant.

The anti-aging effect of *G. lucidum* on mice is comprehensive, and, overall, in addition to regulating the brain, it also improves the damage of various organs and common aging indicators. In this study, compared to the control group, Oil red O staining of the liver in the TGL-treated group was significantly better (Figure 2D, *p* < 0.05), and the number of apoptosis cells was reduced (Figure 5B, *p* < 0.05), mTOR pathway is also activated in female liver tissue (Figure 6A, *p* < 0.05). Meanwhile, liver tissue had a longer telomere length in the TGL group (Figure 6B, *p* < 0.05). Brown adipose tissue depot contribute to improving glucose metabolism, weight loss, and reversing insulin resistance, and consumption of excess fat (Villarroya et al., 2017; Singh et al., 2018), TGL significantly improved the brown adipose tissue levels around the scapula, while adipocytes in the control group were greater and larger (Figure 5A). A feature of cellular senescence is the activity of senescence-associated β-galactosidase (SA-β-gal) (Jurk et al., 2012), The β-galactosidase staining of kidney tissues in the control group was significantly higher than that in the TGL-treated group (Figure 5C, *p* < 0.05), which indicated that TGL could reduce the senescence marker β-galactosidase. Accumulation of iron coupled with impaired ferritinophagy and inhibition of ferroptosis, ferritin (iron storage) levels are becoming biomarkers of cellular senescence (Masaldan et al., 2018). Iron metabolism is tightly regulated in organisms, keeping cells in iron homeostasis. Excessive Fe2+ in cells is toxic, which can induce the body to produce large amounts of reactive oxygen species (ROS). This leads to further attack by ROS and also oxidization of lipids in the cell membrane, causing cell death and aging (Cutler et al., 2014). In this study, Prussian blue iron staining was deeper and more extensive in the spleen of the control group than that in the TGL-treated group (Figure 2E, *p* < 0.05); Western blotting assays showed that the expressions of GPX4 and S100A4 were significantly upregulated in the female TGL-treated group (Figure 6C, *p* < 0.05), which indicated that the ferroptosis would be inhibited or delayed by TGL in aging.

### Regulation of Triterpenoids of *G. lucidum* on Sphingolipid Metabolism in Normal Aged Mice

**FIGURE 7:**
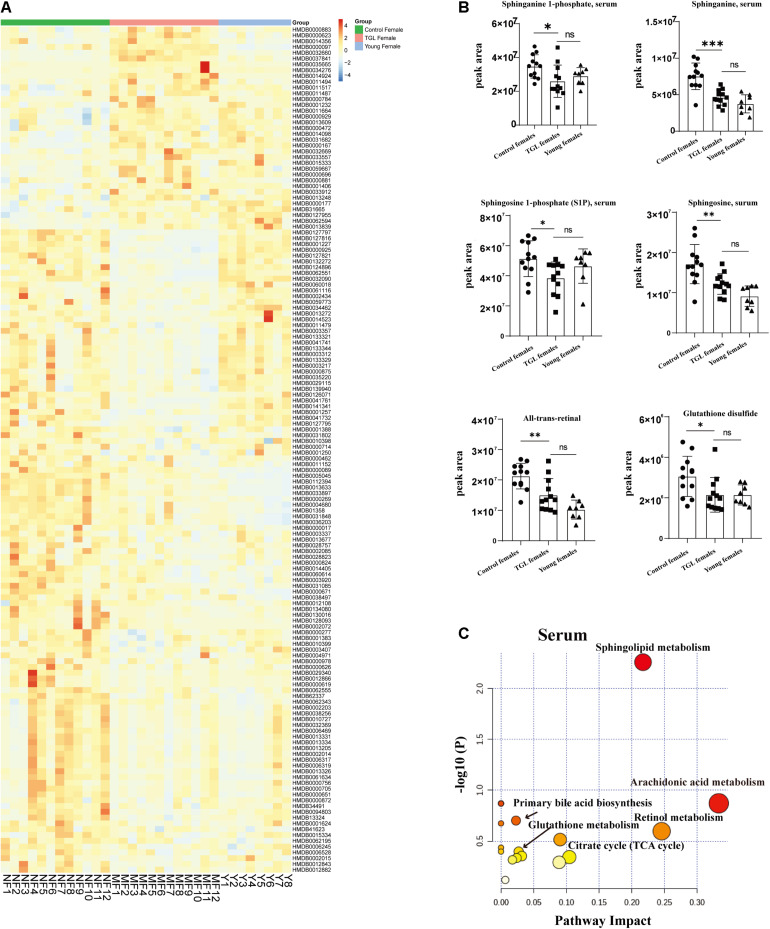
Effect of Triterpenoids of *G. lucidum* on sphingolipid metabolism of normal aged mice. **(A)** Heatmap of serum metabolites; **(B)** Effects on sphingolipid metabolites, including the sphinganine 1-phosphate, sphinganine, sphingosine 1-phoshphate (S1P), sphingosine, all-trans-retinal, and glutathione disulfide; **(C)** results of serum metabolomics using KEGG pathway analysis showed that sphingolipid metabolism and arachidonic acid metabolism are the mainly influenced pathways after treatment of TGL; *n* ≥ 14. *p < 0.05, **p < 0.01, ***p < 0.001, and *ns*, not statistically significant.

Based on the more significant improvements of TGL on female aging mice, to determine whether TGL targeted sphingolipid metabolism to exert its role in delaying aging, metabolomics analysis of serum was performed. At the same time, we added young female C57BL/6 mice (12 weeks) as a reference to observe whether the TGL group tends to the younger group. We found that the serum metabolites were significantly different between the TGL-treated groups and the control group and the young control group (Figure 7A and Supplementary Figures 3A,B). Most sphingolipid metabolites, including sphinganine 1-phosphate, sphinganine, sphingosine 1-phoshphate (S1P), sphingosine, all-trans-retinal, and glutathione disulfide, differed in the TGL-treated groups compared to the control group (Figure 7B, *p* < 0.05), while there were no differences in the TGL-treated groups and young control group (Figure 7B, *p* > 0.05). And metabolites of the brain can also be clearly distinguished between the control group and the TGL group (Supplementary Figures 2C,D). The results of serum and brain metabolism using KEGG pathway analysis showed that sphingolipid metabolism and arachidonic acid metabolism are the mainly influenced pathways (Figure 7C, Supplementary Figure 2E, and Tables 2, 3). Previous studies (Van Liew et al., 1993; Cutler et al., 2014; Hannun and Obeid, 2018; Trayssac et al., 2018; Jesko et al., 2019) indicated that sphingolipid metabolism could regulate development, lifespan, and age-related diseases, and the mTOR signaling pathway could also be modulated by bioactive sphingolipids (Jesko et al., 2019). In this study, we found that phosphorylated-mTOR was activated in the brain (Figure 3C, *p* < 0.05) and liver (Figure 6A, *p* < 0.05) of TGL-treated groups, and especially in females. These results indicated that the TGL may target sphingolipid metabolism to improve the lifespan and age-related diseases, and the mTOR signal pathway.

**TABLE 2:** Metabolites pathway analysis of serum differences metabolites between control group and TGL-treated group.

ID Annotation	Set	In set	Background	In background	*p*-value
Sphingolipid metabolism	39	5	3584	25	5.31E-06
Metabolic pathways	39	24	3584	1455	6.41E-03
Calcium signaling pathway	39	1	3584	10	1.04E-01
Neuroactive ligand-receptor interaction	39	2	3584	128	4.09E-01
Fc gamma R-mediated phagocytosis	39	1	3584	8	8.39E-02
Steroid hormone biosynthesis	39	1	3584	99	6.67E-01
Aldosterone-regulated sodium reabsorption	39	1	3584	8	8.39E-02
Arachidonic acid metabolism	39	2	3584	75	1.96E-01
Fructose and mannose metabolism	39	1	3584	48	4.11E-01
Glycine, serine and threonine metabolism	39	3	3584	49	1.55E-02
Valine, leucine, and isoleucine biosynthesis	39	2	3584	28	3.65E-02
Porphyrin and chlorophyll metabolism	39	2	3584	126	4.01E-01
Aminoacyl-tRNA biosynthesis	39	5	3584	75	1.16E-03
Biosynthesis of secondary metabolites	39	9	3584	1023	8.25E-01
ABC transporters	39	5	3584	90	2.62E-03
Glycerophospholipid metabolism	39	2	3584	46	8.85E-02
Arginine and proline metabolism	39	2	3584	82	2.24E-01
beta-Alanine metabolism	39	2	3584	31	4.40E-02
Glutathione metabolism	39	2	3584	38	6.35E-02
Nicotinate and nicotinamide metabolism	39	2	3584	44	8.20E-02
Valine, leucine and isoleucine degradation	39	1	3584	41	3.63E-01
Propanoate metabolism	39	1	3584	36	3.27E-01
Pantothenate and CoA biosynthesis	39	1	3584	27	2.57E-01
Histidine metabolism	39	1	3584	44	3.84E-01
Riboflavin metabolism	39	1	3584	21	2.06E-01
Retinol metabolism	39	1	3584	24	2.32E-01
Phototransduction	39	1	3584	8	8.39E-02
Tryptophan metabolism	39	5	3584	81	1.64E-03
Phenylalanine, tyrosine and tryptophan biosynthesis	39	1	3584	27	2.57E-01
alpha-Linolenic acid metabolism	39	2	3584	40	6.95E-02
Biosynthesis of unsaturated fatty acids	39	2	3584	54	1.16E-01
Vitamin B6 metabolism	39	1	3584	32	2.96E-01
Purine metabolism	39	1	3584	92	6.39E-01
Phenylalanine metabolism	39	2	3584	46	8.85E-02
Cysteine and methionine metabolism	39	1	3584	56	4.61E-01
Butanoate metabolism	39	1	3584	40	3.56E-01
D-Glutamine and D-glutamate metabolism	39	1	3584	12	1.23E-01

*Use online software MBROLE (version 2.0) (csbg.cnb.csic.es/mbrole2/index.php) for differences metabolites pathway analysis.*

**TABLE 3:** Metabolites pathway analysis of differences metabolites of brain tissue between control group and TGL-treated group.

Pathway	Total Cmpd	Hits	Holm adjust	FDR	Impact
Glycerophospholipid metabolism	36	2	1	0.28258	0.17539
Sphingolipid metabolism	21	1	1	0.69273	0.15416
Lysine degradation	25	1	0.35624	0.10178	0.14085
Terpenoid backbone biosynthesis	18	1	0.005943	0.005943	0.11429
Cysteine and methionine metabolism	33	1	0.14592	0.063101	0.10446
Histidine metabolism	16	1	0.89835	0.14374	0.09016
Purine metabolism	66	3	0.45432	0.10904	0.06093
Pyruvate metabolism	22	1	1	0.19028	0.0591
Glycerolipid metabolism	16	1	1	0.28982	0.04361
Primary bile acid biosynthesis	46	1	0.79466	0.12465	0.02285
Valine, leucine and isoleucine degradation	40	2	1	0.36274	0.02264
Steroid hormone biosynthesis	77	1	0.5312	0.11183	0.02188
Fatty acid biosynthesis	47	1	1	0.44825	0.01473
Aminoacyl-tRNA biosynthesis	48	2	0.17353	0.063101	0
Retinol metabolism	16	1	0.69798	0.12465	0
Taurine and hypotaurine metabolism	8	1	0.79466	0.12465	0
Arachidonic acid metabolism	36	1	1	0.28258	0
Linoleic acid metabolism	5	1	1	0.28258	0
alpha-Linolenic acid metabolism	13	1	1	0.28258	0
Valine, leucine and isoleucine biosynthesis	8	1	1	0.28258	0
Pantothenate and CoA biosynthesis	19	1	1	0.28258	0
Fatty acid elongation	39	1	1	0.44825	0
Fatty acid degradation	39	1	1	0.44825	0
Biosynthesis of unsaturated fatty acids	36	1	1	0.44825	0

### Improvements of TGL on APP/PS1 Mice

**FIGURE 8:**
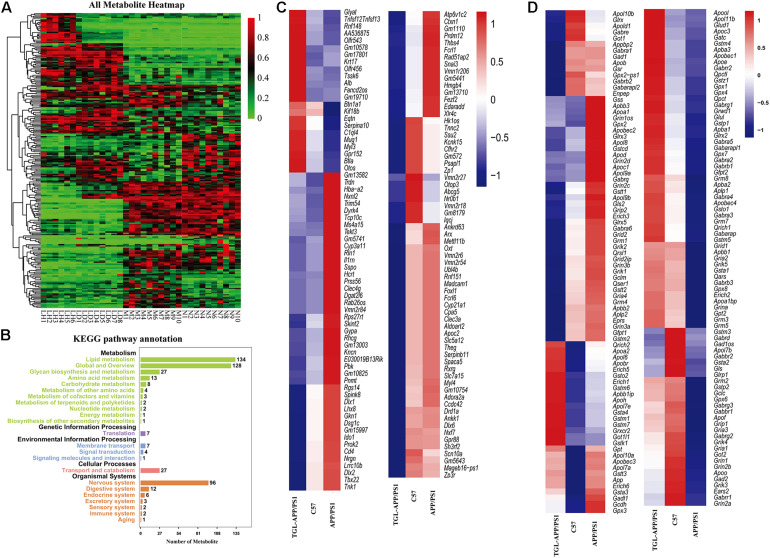
Improvements of triterpenoids of *G. lucidum* in APP/PS1 mice. **(A)** Heatmap of serum metabolites; **(B)** KEGG analysis of serum metabolites; **(C,D)** Different expressed mRNA of brain tissues. More than six independent experiments in Serum metabolomics and more than three independent experiments in Transcriptome sequencing.

To further evaluate improvement by TGL on aging, and to verify the pathway of TGL improving brain injury, we performed a combined analysis of metabolomics and transcriptome data of TGL-treated APP/PS1 mice. The metabolites present in the serum of TGL-treated APP/PS1 mice had changed (Figure 8A), and the KEGG pathway analysis of these metabolites showed that there were about 117 metabolites enriched in glycerophospholipid metabolism, 27 metabolites enriched in glycosylphosphatidylinositol (GPI)-anchor biosynthesis, and 2 metabolites enriched in the sphingolipid metabolism and the sphingolipid signaling pathway (see Figure 8B for additional details).

RNA sequencing of global brain tissues indicated that most mRNAs expressed belonged to genes involved in the secretion and metabolism of neurotransmitters (Figures 8C,D) and ion channel for potassium, sodium, and calcium (Figures 8C,D), and this involved genes in synaptic regulation (Figures 8C,D), histone modification (Figures 8C,D), and electrolytes regulation (Figures 8C,D), which recovered to levels similar to those expressed in healthy controls. KEGG enrichment analysis of the differentially expressed mRNA sequences showed similar patterns to that of a previously published paper (Cutler et al., 2014). The IPA (Ingenuity Pathway Analysis) analysis indicated that the function and diseases, including carbohydrate metabolism and lipid metabolism, were influenced (Supplementary Table 4) and that differentially expressed mRNAs were enriched for the sphingolipid metabolism pathway (Supplementary Table 5). The above evidence indicated that TGL exerted multi-system, multi-target, multi-directional comprehensive regulation on the brain, including improvement in metabolism, immunity, neurotransmitters, electrolyte balance, synaptic transmission, and histone methylation. Together with our previous study that alcohol extracts of *G. lucidum* could improve the system of AD (Lai et al., 2019), all of this demonstrates that TGL has multiple biological activities that could target the gut–brain axis to stimulate the CNS in a manner that regulates host metabolism, the immune response, and other key signaling pathways.

### Ganodenic Acid A Improved Regulation of Sphingolipid Metabolism in 3 × Tg-AD Mice

**FIGURE 9:**
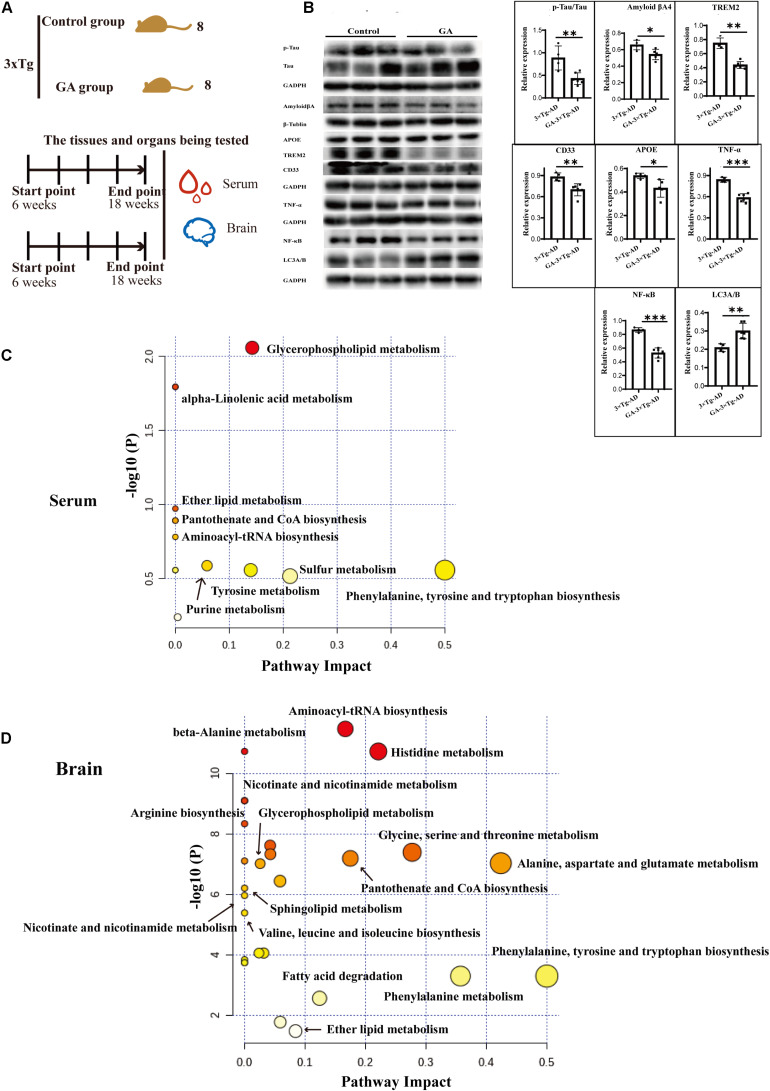
Ameliorate effects of Ganodenic acid A by regulating sphingolipid metabolism in 3 × Tg-AD mice. **(A)** Experimental procedure in 3 × Tg-AD mice; **(B)** The AD biomarkers of p-Tau, Aβ, APOE, TREM2, CD33, the inflammatory cytokines of TNF-α and NF-κB p65 and the autophagy level of LC3A/B were measured in brain tissues; **(C)** KEGG analysis of serum metabolites; **(D)** KEGG analysis of brain metabolites. Data are presented as the means ± SD of more than six independent experiments and six independent experiments in Western blot. *p < 0.05, **p < 0.01, and ***p < 0.001.

Based on previous pharmacological investigations of *G. lucidum* (Kalvodova et al., 2005; Wang et al., 2007; Maceyka et al., 2012; Teekachunhatean et al., 2012; Cheng et al., 2013; Guo et al., 2013; Miura et al., 2014; Hirano-Sakamaki et al., 2015; Chakrabarti et al., 2016; Cao et al., 2017; Lin et al., 2017; MOW Grimm et al., 2017; Pera et al., 2017; Angelopoulou and Piperi, 2019; Fabiani and Antollini, 2019; Jesko et al., 2019; Kurz et al., 2019; Magistretti et al., 2019; Crivelli et al., 2020), as shown in Figure 9A, after the 10 weeks treatment of TGL, serum and brain tissue were used to evaluate the regulation of *Ganoderma* triterpenes on sphingolipid metabolism. First, the expression of AD biomarkers p-Tau, β-amyloid (Aβ) peptides, APOE, TREM2, CD33 in brain tissues were reduced (Figure 9B, *p* < 0.05), the inflammatory cytokines of TNF-α and NF-κB p65 were inhibited (Figure 9B, *p* < 0.05), and the level of the autophagy-associated gene LC3A/B was upregulated (Figure 9B, *p* < 0.05). These results indicated that ganodenic acid A might be the effective constituent improving the systems of AD.

Next, an unsupervised metabolome analysis of serum and brain samples was used to define the mechanism of action involved in ganodenic acid A activity. The analysis showed that glycerophospholipid metabolism, sphingolipid metabolism, pantothenate, and CoA biosynthesis were influenced (Figures 9C,D) by exposure to ganodenic acid A, which indicated that it is active in sphingolipid metabolism to reduce the biomarkers of AD.

## Discussion

Our data provide further evidence that long-term administration of TGL could mitigate the age-associated physiological decline in the normal aging mice, including cataract formation, hair loss and skin relaxation, brown adipose tissue accumulation, the β-galactosidase staining degree of the kidney, and the iron death of spleen and brain function. Testing using the APP/PS1 mice model showed that TGL could also improve brain function in AD (Lai et al., 2019), and measurements in the 3 × Tg-AD mice showed that ganoderic acid A might be the effective constituent involved in delaying brain aging in AD. A likely mechanism of brain improvement may involve the regulation of sphingolipid metabolism, prolonging telomere length, enhancing autophagy to remove pathological metabolites.

Although the relationships between telomere shortening, aging, and disease are still not entirely clear, it can be affirmed that normal aging is dependent on telomere shortening (Blackburn et al., 2015). Studies have demonstrated that a reduction in telomere length leads to the cessation of cell division; thus cellular senescence, apoptosis, exposure to antioxidants, and anti-inflammatory activity can attenuate the degree of shortening of telomere length during aging (Blackburn et al., 2015). In this study, we found that telomere lengths in the liver and brain in the TGL-treated groups were longer than in the normal group, which indicated that TGL could slow the telomere shortening to anti-aging and reduce susceptibility to cancer.

The results in this study showed that TGL had effects on delaying aging, and the TGL-treated female group generally showed a more obvious improvement concerning all these aging-associated changes. Unsupervised metabolomics analysis showed that different sphingolipid metabolites were present in serum and brain samples in normal aged mice following TGL treatment. The relative KEGG pathway analysis indicated that the sphingolipid metabolism and glycerophospholipid metabolism pathways were enriched (Figure 6 and Table 2), which indicated that the TGL could target sphingolipid metabolism. Sphingolipid metabolism and glutathione metabolism play important roles in senescence and age-related diseases (Maher, 2005; Trayssac et al., 2018). Sphingolipids are a highly conserved class of lipids. They were discovered in the brain more than a century ago, and are known as cerebral glycosides (Yeh, 1984; Sonnino and Prinetti, 2016). Members of this diverse and ubiquitous lipid family share a common structural feature of a long chain backbone skeleton and are important components of the lipid bilayer, where they contribute to the structure and function of membranes, the interconversion of vesicle transport, and membrane dynamics (Olsen and Faergeman, 2017). Ceramide is the central molecule of all sphingomyelin metabolism (Brodowicz et al., 2018). Its formation begins with the condensation of two common cellular metabolites, serine and palmitoyl CoA, in the endoplasmic reticulum (ER). The other metabolites of the products are 3-ketosphingominol, dihydro-sphingominol (known as sphingominol), and glycolipids (GSL), including gangliosides containing sialic acid and lactosylceramides (LacCer), ceramide 1-phosphate, and more complex sphingolipids, such as GSL and sphingolipids. Increasing studies have shown these membrane sphingolipids, composed of sphingolipids and ceramides and sphingolipases and its enzymes, exert important roles in signal transduction, especially in regulating the central nervous system (CNS) physiology (Yeh, 1984; Hirabayashi, 2012; Assi et al., 2013; Sonnino and Prinetti, 2016; Olsen and Faergeman, 2017; Brodowicz et al., 2018; Vutukuri et al., 2018; Hussain et al., 2019). In our study, levels of sphingolipid metabolites, including the sphinganine 1-phosphate, sphinganine, S1P, sphingosine, all-trans-retinol, and glutathione disulfide, were altered in the TGL-treated groups and the control group of the normal-aged mice (Figure 6). Retinol metabolism has been associated with an increased risk of cataract development (Nourmohammadi et al., 2008), our analysis showed that TGL could reduce the incidence rate of cataracts.

Over the past few decades, studies have revealed that abnormal sphingolipid metabolism is involved in the pathology of AD (Dinkins et al., 2016; Martinez and Mielke, 2017; Toledo et al., 2017; Czubowicz et al., 2019). The increased *de novo* synthesis of ceramide accelerated neuronal differentiation, while high levels of ceramide were detected in AD and other neurodegenerative diseases (Lin et al., 2017). As ceramide levels in cerebrospinal fluid and white matter are also elevated, especially in the milder symptoms of AD dementia (Czubowicz et al., 2019; Kurz et al., 2019), several other clinical studies have revealed that plasma ceramide levels are associated with mild cognitive impairment (MCI) and memory loss in AD patients. High plasma ceramide levels have been associated with hippocampal volume loss, thus the changes in brain and plasma ceramide levels could be used as a biomarker of the early stages of AD (Chakrabarti et al., 2016; Dinkins et al., 2016; MOW Grimm et al., 2017).

Other potential targets of ceramide metabolism, such as sphingomyelin, sphingomyelin, sphingomyelinase, and sphingosine kinase 1 (SK1), have also been identified (Martinez and Mielke, 2017; Olsen and Faergeman, 2017; Hussain et al., 2019). Studies have shown that higher levels of sphingolipids can inhibit the γ-secretase activity during amyloid precursor protein (APP) decomposition, thereby reducing the accumulation of Aβ (Pera et al., 2017; Fabiani and Antollini, 2019). Ceramide, in turn, stabilizes BACE-1, which helps cleave the Aβ (Kalvodova et al., 2005). As ceramides can be converted to sphingosine, levels of sphingosine are also increased in the brain of patients with AD. Since sphingosine can be phosphorylated to form S1P (which is reduced in AD), this pathway may be of interest in studying potential pharmacological targets for AD (Maceyka et al., 2012; Czubowicz et al., 2019). Gilenya is an S1P receptor agonist approved for the treatment of multiple sclerosis and has been shown to modulate the neuroinflammatory pathway in mice with AD (Angelopoulou and Piperi, 2019; Jesko et al., 2019; Crivelli et al., 2020). Since almost all nerve cells express the S1P receptor, it has been proposed as an interesting target for drug treatment of AD (Angelopoulou and Piperi, 2019; Jesko et al., 2019; Crivelli et al., 2020). More complex changes in GSL were observed in AD, and high GM1 levels appear to be associated with Aβ. Specifically, the ganglioside GM1 was enriched in lipid rafts, leading to the toxic accumulation and aggregation of Aβ (Miura et al., 2014; Hirano-Sakamaki et al., 2015; Magistretti et al., 2019), suggesting that there was a strong association between the steady-state ganglioside levels and AD. In this study, we found that the sphingolipid metabolites, including the sphinganine-1-phosphate, sphinganine, S1P, sphingosine, and all-trans-retinal and glutathione disulfide, were altered in TGL-treated groups and control groups in the APP/PS1 and 3 × Tg-AD mice models (Figures 7, 8). Furthermore, ganoderic acid A could be detected in the brain after intravenously dosing using UFLC-MS/MS, which suggested triterpenoids could cross the blood–brain barrier (Supplementary Table 2; Cao et al., 2017). In addition, considering that *G. lucidum* also exerts different pharmacological activities in the heart, liver, spleen, and brain (Supplementary Tables 2, 3), this indicates that TGL may improve brain function likely by delaying the brain aging in AD through the regulation of sphingolipid metabolism, and ganoderic acid A might represent active constituent.

### Limitation of the Study

Because of the long period required to obtain the normal aged mice and the COVID-19 pandemic, we failed to complete the survival curve experiment, so we cannot fully determine whether triterpenoids of *G. lucidum* can prolong the life span. Due to the complexity of the chemical constituents of *G. lucidum* and the high costs of isolation and purification of single components, the present and previous studies have mostly focused on the study of mixtures. Thus, it is difficult to conduct in-depth conduct investigations into the pharmacological mechanisms involved. In this study of potential pharmacological activities of triterpenoids from *G. lucidum*, behavioral experiments were performed only in APP/PS1 mice (Lai et al., 2019), and only the effects of ganoderic acid A have been evaluated in the 3 × Tg-AD mouse model.

## Data Availability Statement

The datasets presented in this study can be found in online repositories. The names of the repository/repositories and accession number(s) can be found in the article/Supplementary Material.

## Ethics Statement

The animal protocols used in this study were approved by the Institutional Animal Care and Use committee of the Center of Laboratory Animals of the Guangdong Institute of Microbiology.

## Author Contributions

MZe, LQ, YG, XT, and XZ carried out most of the experiment. MZe, YG, LQ, XT, and XZ perfomed the histopathology and molecular biology experiments. LQ completed the metabolomics-related experiments. YX, QW, DC, and MZh led the progress of the whole experiment and reviewed the manuscript. All authors designed the study, wrote the manuscript, and read and approved the final manuscript.

## Conflict of Interest

The authors declare that the research was conducted in the absence of any commercial or financial relationships that could be construed as a potential conflict of interest.

## Figures and Tables

**Table:** 

Antibodies

Reagent or Resource	Source	Identifier	Working concentration (primary antibodies)	Official website
GAPDH	ProteinTech	60004-1-Ig	1:10000	http://www.ptgcn.com/
mTOR	ProteinTech	20657-1-Ap	1:1000	http://www.ptgcn.com/
Phospho-mTOR (Ser2448)	Affinity	AF3308	1:1000	http://www.affbiotech.com/
LC3A/B	Abcam	ab128025	1:1000	https://www.abcam.com/
Goat-Anti-Rabbit IgG (H+L)HRP	Affinity	S0001	1:10000	http://www.affbiotech.com/
Goat-Anti-Mouse IgG (H+L)HRP	Affinity	S0002	1:10000	http://www.affbiotech.com/
GPX4	Affinity	DF6701	1:1000	http://www.affbiotech.com/
S100A4	Affinity	S100A4	1:1000	http://www.affbiotech.com/
p-Tau	Affinity	AF3308	1:1000	http://www.affbiotech.com/
Tau	Abcam	Ab32057	1:1000	https://www.abcam.com/
Amyloid β	Affinity	AF6084	1:1000	https://www.abcam.com/
APOE	Abcam	Ab204112	1:1000	https://www.abcam.com/
TREM2	Affinity	DF12529	1:1000	http://www.affbiotech.com/
CD33	Affinity	DF6789	1:1000	http://www.affbiotech.com/
TNF-α	Abcam	ab9579	1:1000	https://www.abcam.com/
NF-κB	Abcam	ab16502	1:1000	https://www.abcam.com/

**Chemicals, Peptides, and Recombinant Proteins**

**Reagent or Resource**			**Source**	**Identifier**

RNAiso Plus			Takara	9109
DNAiso Reagent			Takara	9770Q

**Critical Commercial Assays**

**Reagent or Resource**		**Source**	**Identifier**	**Official website**

Detection kit for β-galactosidase (β-Gal) activity		Solarbio	BC2580	http://www.solarbio.com/
TMR (Red) Tunel Cell Apoptosis Detection Kit		Servicebio	G1502	https://www.servicebio.cn/
TB Green Premix Ex Taq II (Tli RNaseH Plus)		Takara	RR820A	https://www.takarabiomed.com.cn/
Prussian blue staining kit		Servicebio	G1029	http://www.servicebio.cn/
Relative Mouse Telomere Length Quantification qPCR Assay Kit		ScienCell	#M8908	https://www.sciencellonline.com/

**Deposited Data**				

RNA sequencing data		This paper		PRJNA669847
		

## Footnotes

1 https://www.thermofisher.com/cn/zh/home.html

2 http://www.affbiotech.com/

3 http://www.genome.jp/kegg/

## Supplementary Material

Supplementary Figure 1 The specific content of each compound in triterpenoids of *G. lucidum*. **(A)** Separation and purification process of triterpenoids of *G. lucidum*; **(B)** High-performance liquid chromatography (HPLC) of triterpenoids of *G. lucidum*.

Supplementary Figure 2 Effect of Triterpenoids of *G. lucidum* on hypothalamus RNA sequencing of normal aged mice. Gene Ontology (GO) analysis **(A)** Cellular component; **(B)** Biological process; **(C)** Molecular function.

Supplementary Figure 3 Effect of triterpenoids of *G. lucidum* on serum and brain metabolism of normal aged mice. **(A)** Hierarchical Clustering Heatmaps of serum metabolite (Distance Measure: Euclidean; Clustering Algorithm: Ward); **(B)** Principal Component Analysis (PCA) of serum metabolite; **(C)** Hierarchical Clustering Heatmaps of brain tissue metabolite (Distance Measure: Euclidean; Clustering Algorithm: Ward); **(D)** Principal Component Analysis (PCA) of brain tissue metabolite; **(E)** Metabolites pathway analysis of brain tissue differences metabolites between the control group and TGL-treated group. Use online software MetaboAnalyst (version 4.0) (http://www.metaboanalyst.ca/) for differences in metabolites pathway analysis; *n* ≥ 14.

Supplementary Table 1 The main active substance of triterpenoids of *G. lucidum*.

Supplementary Table 2 Pharmacokinetic parameters of triterpenes of *G. lucidum*. Tmax, time to peak; Cmax, Peak blood concentration; Vd, apparent volume of distribution; t1/2α, Distribution half-life; t1/2β, Elimination half-time; ig, intragastric administration; iv, intravenous immunoglobulin; per os, oral administration; #, mL min^–1^ kg^–1^; ^∗^, L h^–1^ kg^–1^.

Supplementary Table 3 *G. lucidum* and its effects (Phu et al., 2020).

Supplementary Table 4 The IPA analysis showed that the function and diseases, including carbohydrate and lipid metabolism, were influenced (APP/PS1).

Supplementary Table 5 The different expressed mRNAs enriched into the sphingolipid metabolism (APP/PS1).
