# Supplementary figures and images for: Long-Term Administration of Triterpenoids From Ganoderma lucidum Mitigates Age-Associated Brain Physiological Decline via Regulating Sphingolipid Metabolism and Enhancing Autophagy in Mice
Source: Front Aging Neurosci. 2021 May 6;13:628860. doi: 10.3389/fnagi.2021.628860 (PMC8134542; doi:10.3389/fnagi.2021.628860)

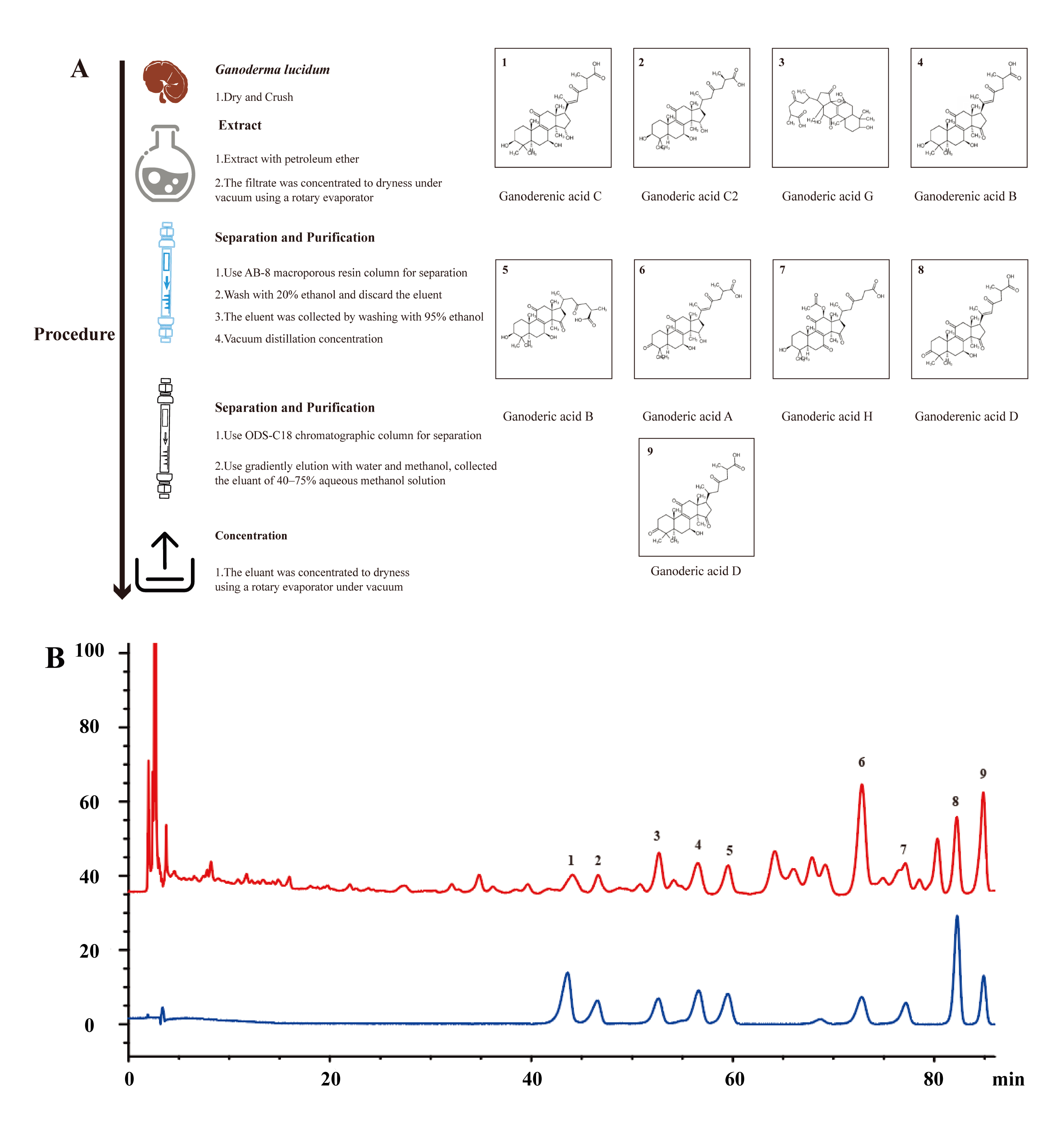

Supplement: Supplementary Figure 1 — The specific content of each compound in triterpenoids of G. lucidum. (A) Separation and purification process of triterpenoids of G. lucidum; (B) High-performance liquid chromatography (HPLC) of triterpenoids of G. lucidum. [file Image_1.TIF]

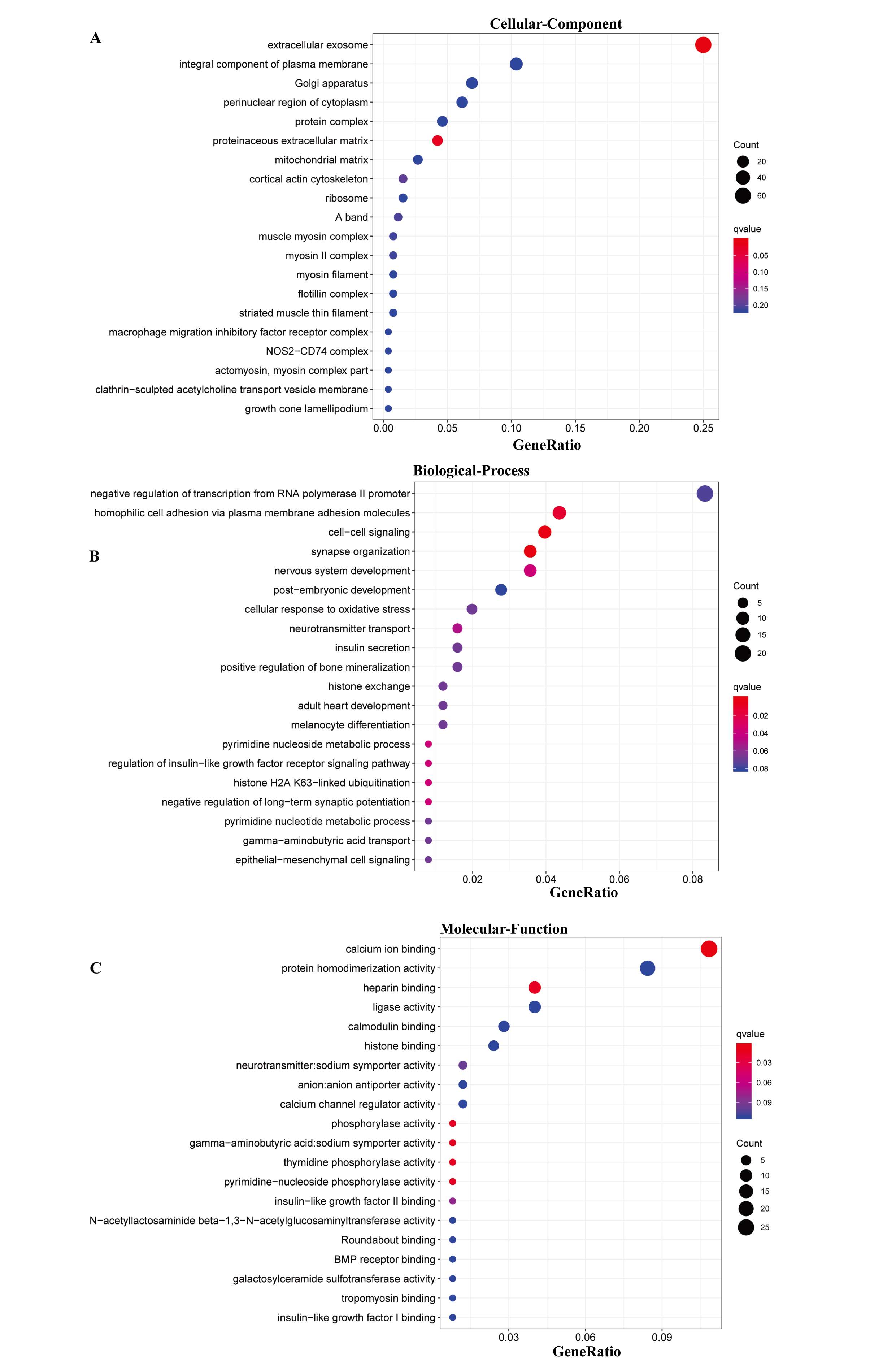

Supplement: Supplementary Figure 2 — Effect of Triterpenoids of G. lucidum on hypothalamus RNA sequencing of normal aged mice. Gene Ontology (GO) analysis (A) Cellular component; (B) Biological process; (C) Molecular function. [file Image_2.TIF]

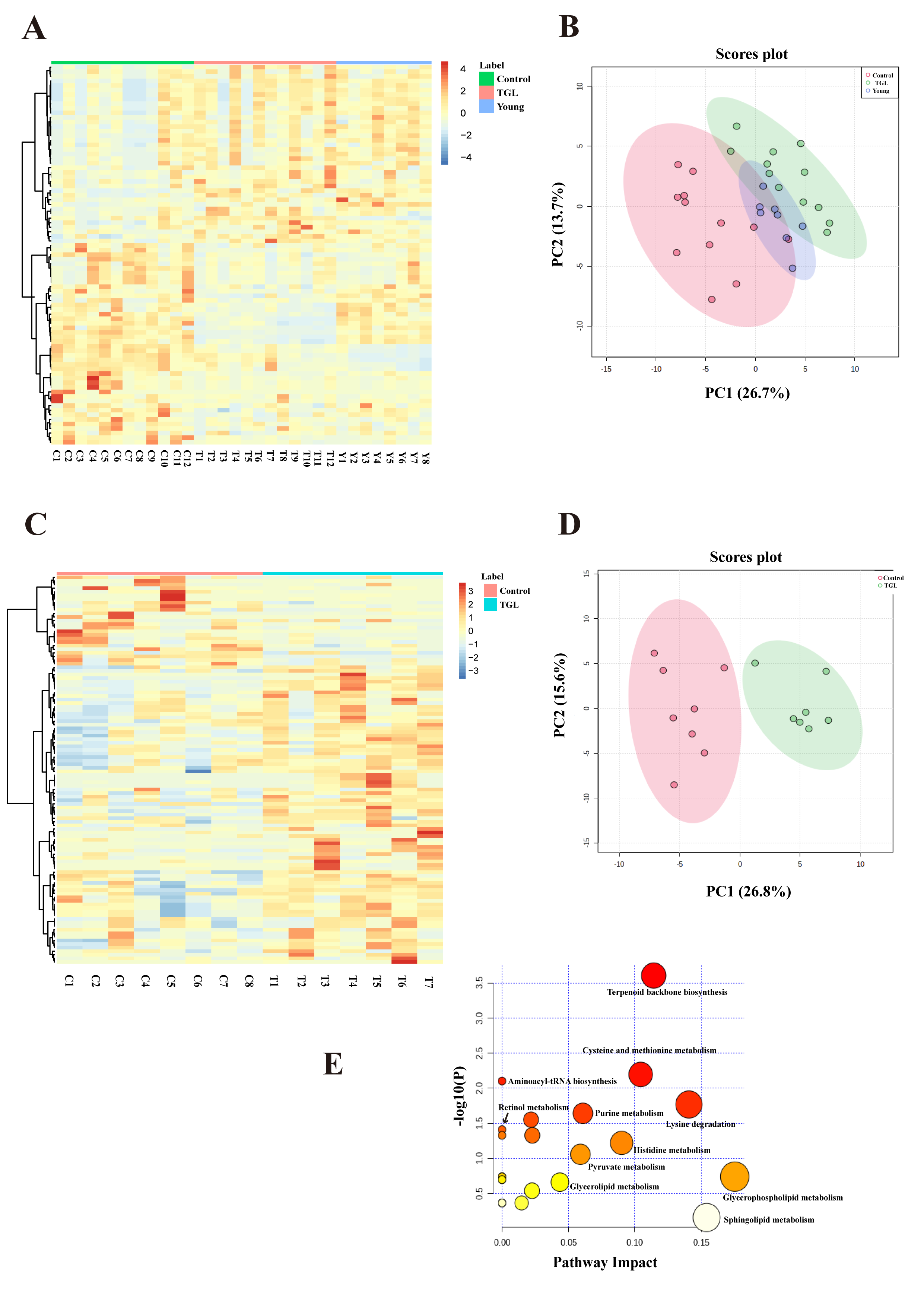

Supplement: Supplementary Figure 3 — Effect of triterpenoids of G. lucidum on serum and brain metabolism of normal aged mice. (A) Hierarchical Clustering Heatmaps of serum metabolite (Distance Measure: Euclidean; Clustering Algorithm: Ward); (B) Principal Component Analysis (PCA) of serum metabolite; (C) Hierarchical Clustering Heatmaps of brain tissue metabolite (Distance Measure: Euclidean; Clustering Algorithm: Ward); (D) Principal Component Analysis (PCA) of brain tissue metabolite; (E) Metabolites pathway analysis of brain tissue differences metabolites between the control group and TGL-treated group. Use online software MetaboAnalyst (version 4.0) (http://www.metaboanalyst.ca/) for differences in metabolites pathway analysis; n ≥ 14. [file Image_3.TIF]
