# Supplementary material for: Long-Term Administration of Triterpenoids From Ganoderma lucidum Mitigates Age-Associated Brain Physiological Decline via Regulating Sphingolipid Metabolism and Enhancing Autophagy in Mice
Source: Front Aging Neurosci. 2021 May 6;13:628860. doi: 10.3389/fnagi.2021.628860 (PMC8134542; doi:10.3389/fnagi.2021.628860)
Supplement: Supplementary Table 1 — The main active substance of triterpenoids of G. lucidum. [file Table_1.pdf]

## Supplementary Tables

## Table S1 The main active substance of triterpenoids of G. lucidum

| Compound | CAS Number | Molecular formula | Molecular weight | Extract content (triterpenoids per g of feed) (mg/g) |
|---|---|---|---|---|
| Ganoderenic acid C | 100665-42-7 | $C_{30}H_{44}O_7$ | 516.666 | 0.00017 |
| Ganoderic acid C2 | 103773-62-2 | $C_{30}H_{46}O_7$ | 518.682 | 0.00025 |
| Ganoderic acid G | 98665-22-6 | $C_{30}H_{44}O_8$ | 532.666 | 0.00061 |
| Ganoderenic acid B | 100665-41-6 | $C_{30}H_{42}O_7$ | 514.65 | 0.00021 |
| Ganoderic acid B | 81907-61-1 | $C_{30}H_{44}O_7$ | 516.666 | 0.00045 |
| Ganoderic acid A | 81907-62-2 | $C_{30}H_{42}O_7$ | 514.65 | 0.00156 |
| Ganoderic acid H | 98665-19-1 | $C_{31}H_{42}O_9$ | 558.66 | 0.00098 |
| Ganoderenic acid D | 100665-43-8 | $C_{30}H_{40}O_7$ | 512.634 | 0.00036 |
| Ganoderic acid D | 108340-60-9 | $C_{30}H_{42}O_7$ | 514.65 | 0.00102 |

# Table S2 Pharmacokinetic parameters of triterpenes of G. lucidum

| Object of study | Triterpenoids | Dosage of administration | Administration route | Absorption parameters | | | Distribution parameter | Elimination parameter | | |
|---|---|---|---|---|---|---|---|---|---|---|
| | | | | $T_{max}$ | $C_{max}$ | AUC | $V_d$/L·kg$^{-1}$ | $t_{1/2\alpha}$ | $t_{1/2\beta}$ | CL |
| SD rat (Cao et al., 2017) | Ganoderic acid A | 20 mg·kg$^{-1}$ | ig | (0.15±0.03)h | (0.91±0.57) μmol·L$^{-1}$ | (1.35±0.46) μmol·h·L$^{-1}$ | 106.24±26.42 | | (2.46±0.75)h | 32.38±13.45* |
| | | 20 mg·kg$^{-1}$ | iv | | | (14.34±4.54) μmol·h·L$^{-1}$ | 10.21±3.47 | | (2.40±0.35)h | 2.91±0.81* |
| | | 20 mg·kg$^{-1}$(brain microdialysis) | iv | (0.25±0) h | (0.61±0.18) μmol·L$^{-1}$ | (0.45±0.09)μmol·h·L$^{-1}$ | 190.61±146.36 | | (1.40±0.93)h | 89.21±16.21* |
| SD rat (Cheng et al., 2013) | Ganoderic acid D | 15 mg·kg$^{-1}$ | iv | | (3582.93±888.57) μg·L$^{-1}$ | ( 2320.18±221.44) μg·h·L$^{-1}$ | 4.42±0.47 | | (0.92±0.02)h | 72.36±6.57# |
| | | 15 mg·kg$^{-1}$ | ig (Conventional solution) | | (107.18±4.84)μg·L$^{-1}$ | (550.64±23.09) μg·h·L$^{-1}$ | 18.51±0.72 | | (2.05±0.08)h | 303.11±12.78# |
| | | 15 mg·kg$^{-1}$ | ig (Nano preparation) | | (1555.59±237.56)μg·L$^{-1}$ | (1629.29±186.23) μg·h·L$^{-1}$ | 6.53±0.45 | | (1.87±0.12)h | 103.25±10.12# |
| | Ganoderic acid B (the metabolite of Ganoderic acid D) | 15 mg·kg$^{-1}$(Ganoderic acid D) | iv | | (998.76±108.72)μg·L$^{-1}$ | (2765.89±483.61) μg·h·L$^{-1}$ | 3.79±0.78 | | (1.62±0.46)h | 61.98±13.10# |
| | | 15 mg·kg$^{-1}$(Ganoderic acid D) | ig (Conventional solution) | | (271.06±18.02)μg·L$^{-1}$ | (1643.90±92.28) μg·h·L$^{-1}$ | 6.38±0.41 | | (4.12±0.14)h | 101.59±5.69# |
| | | 15 mg·kg$^{-1}$(Ganoderic acid D) | ig (Nano preparation) | | (883.95±35.74)μg·L$^{-1}$ | (1900.99±39.53) μg·h·L$^{-1}$ | 6.68±0.26 | | (3.10±0.13)h | 87.80±1.81# |
| SD rat (Guo et al., 2013) | Ganoderic acid C2 | 20 mg·kg$^{-1}$ | per os | 25.19 min | 145.42 μg·L$^{-1}$ | 56600.12 μg·min·L$^{-1}$ | | 3.912 | 213.5607min | |
| SD rat (Wang et al., 2007) | Ganoderic acid C2 | 55.3 mg·kg$^{-1}$ | ig | 16.79 min | 5.23 mg·L$^{-1}$ | 1125.29 mg·min·L$^{-1}$ | 4.85 | 11.61 | 376.08 min | 47.9# |
| | Ganoderic acid B | 258.0 mg·kg$^{-1}$ | ig | 6.26 min | 13.15 mg·L$^{-1}$ | 5771.93 mg·min·L$^{-1}$ | 18.03 | 37.63 | 852.59 min | 44.7# |
| | Ganoderic acid K | 75.8 mg·kg$^{-1}$ | ig | 32.10 min | 2.86 mg·L$^{-1}$ | 923.59 mg·min·L$^{-1}$ | 15.99 | 32.26 | 697.48 min | 82.1# |
| | Ganoderic acid H | 155.0 mg·kg$^{-1}$ | ig | 24.88 min | 4.92 mg·L$^{-1}$ | 986.00 mg·min·L$^{-1}$ | 14.77 | 17.13 | 293.75 min | 157.2# |
| Human (Teekachunhatean et al., 2012) | Ganoderic acid A | (4253.4±122.22) μg | per os (abrosia) | (0.54±0.18)h | (10.99±4.02) μg·L$^{-1}$ | (10.53±4.32) μmin·L$^{-1}$ | | (0.62±0.17)h | | |
| | | (4253.4±122.22) μg | per os(no abrosia) | (1.67±0.88)h | (3.84±1.56) μg·L$^{-1}$ | (11.02±5.54) μmin·L$^{-1}$ | | (1.34±0.65)h | | |
| | Ganoderic acid F | (672.45±24.06) μg | per os (abrosia) | (0.52±0.13)h | (2.57±0.91) μg·L$^{-1}$ | (2.42±0.93) μmin·L$^{-1}$ | | (0.48±0.22)h | | |
| | | (672.45±24.06) μg | per os(no abrosia) | ND | ND | ND | | ND | | |

Note: Tmax, time to peak; Cmax, Peak blood concentration; Vd, apparent volume of distribution; t1/2α, Distribution half-life; t1/2β, Elimination half-time; ig, intragastric administration; iv, intravenous immunoglobulin; per os, oral administration; #, mL·min$^{-1}$·kg$^{-1}$; *, L·h$^{-1}$·kg$^{-1}$

## Table S3 G. lucidum and its effects (Phu et al., 2020)

| Effects | Bioactive Components | Potential Mechanisms | Models | References |
|---|---|---|---|---|
| Anti-aging | Total water extract of G. lucidum | Improves the resistance to oxidative stress via the mTOR/S6K signaling | Caenorhabditis elegans | (Cuong et al., 2019) |
| | G. lucidum ethanol extract | Increases the expression of Nrf2/ HO-1 | C2C12 mouse myoblast cell line | (Lee et al., 2016) |
| | Ganodermanontriol | Increases the expression of Nrf2/ HO-1 via PI3K/Akt | Hepa1c1c7 cells | (Ha et al., 2013) |
| | Ganodermanondiol | Increases the expression of Nrf2/ HO-1 via AMPK | HepG2 cells | (Li et al., 2013) |
| | G. lucidum polysaccharides (RF3) | Activates the expression of DAF-16 via TIR-1 receptor and MAPK | Caenorhabditis elegans | (Chuang et al., 2009) |
| Cognitive impairments | G. lucidum aqueous extract | Inhibits the apoptotic-associated signaling pathways JNK-c-Jun, p38MAP kinase signaling | Cortical neurons | (Lai et al., 2008) |
| | G. lucidum aqueous extract | Up-regulates MAP kinase and cAMP-response element binding protein (CREB) signaling pathways | rat PC12 cells | (Cheung et al., 2000) |
| | G. lucidum ethanol extract | Regulate DNA methylation in Rodents | D-galactose induced Sprague-Dawley rats; APP/PS1 mice; SAMP8 mice | (Lai et al., 2019) |
| Hypoglycemic effects | G. lucidum polysaccharides (containing 89% total carbohydrate and 11% uronic acid.) | Decreases the expression of PEPCK | Obese/diabetic (+db/+db) mice | (Liang et al., 2018) |
| | G. lucidum polysaccharides | Decreases the mRNA expression of hepatic glycogen phosphorylase, glucose-6-phosphatase, fructose-1,6-bisphosphatase | Type 2 diabetic mice | (Xiao et al., 2012) |
| | G. lucidum polysaccharides (F31) | Decreases the mRNA levels of hepatic glucose regulatory enzymes via AMPK activation | Type 2 diabetic mice | (Xiao et al., 2017) |
| | G. lucidum polysaccharides | Facilitates $Ca2^{+}$ entry into pancreatic β cells beta cells | Normal fasted mice | (Zhang et al., 2004) |
| | GLPs proteoglycan extract (FYGL) | Suppress the expression of protein tyrosine phosphatase 1B (PTP1B) | Type 2 diabetes mellitus(T2DM) rats. | (Teng et al., 2012) |
| | GLPs proteoglycan extract (FYGL) | Inhibits the expression of PTP1B, activate PI3K/Akt increases phosphorylation of AMPK, up-regulate the expression of GLUT 4 | Obese C57BL/6(ob/ob) mice, rat myoblast L6 cells | (Yang et al., 2018) |
| Antihyperlipidemic effects | G. lucidum spores | Up-regulates acyl-CoA oxidase 1 (Acox1) and Insig-1/2 gene expression | Diabetic rats | (Wang et al., 2015) |
| | G. lucidum ethanol extract (GL95) | Reduces the mRNA levels of FAS, ACAT2, SREBP-1C, HMGCR elevates the mRNA levels of CYP7A1, PPARα, ApoB and Acox1 | HFD-fed Wistar rats | (Guo et al., 2018) |
| | G. lucidum ethanol extract | Activates leptin-mediated signaling to improve metabolic regulation | HFD-fed mice | (Diling et al., 2020) |
| Antitumous effect | Ganoderic acid A/DM | Induced NDRG2 over-expression | In vitro cell culture and in vivo cell-line-derived orthotopic xenograft animal models of anaplastic meningioma | (Das et al., 2020) |

**Table S4 The IPA analysis showed that the function and diseases, including the Carbohydrate Metabolism, Lipid Metabolism, were influenced (APP/PS1).**

| Category | p-value | Category | p-value |
|---|---|---|---|
| Cancer | 1.49E-29-1.69E-04 | Nervous System Development and Function | 6.47E-08-1.91E-05 |
| Dermatological Diseases and Conditions | 1.49E-29-1.8E-04 | Hematological Disease | 1.44E-07-1.61E-04 |
| Organismal Injury and Abnormalities | 1.49E-29-1.89E-04 | Auditory Disease | 1.44E-07-1.44E-07 |
| Neurological Disease | 3.5E-24-1.89E-04 | Developmental Disorder | 3.3E-07-1.76E-04 |
| Gastrointestinal Disease | 1.86E-23-1.83E-04 | Cellular Movement | 3.81E-07-1.6E-04 |
| Hereditary Disorder | 4.26E-21-5.65E-05 | Hematological System Development and Function | 3.81E-07-1.6E-04 |
| Psychological Disorders | 4.26E-21-1.73E-04 | Immune Cell Trafficking | 3.81E-07-1.6E-04 |
| Skeletal and Muscular Disorders | 4.26E-21-1.44E-04 | Cell Signaling | 4.61E-07-1E-04 |
| Cardiovascular Disease | 1.64E-13-1.73E-04 | Vitamin and Mineral Metabolism | 4.61E-07-6.88E-06 |
| Nutritional Disease | 8.43E-13-9.04E-09 | Nucleic Acid Metabolism | 7.47E-07-1E-04 |
| Endocrine System Disorders | 1.46E-12-1.69E-04 | Small Molecule Biochemistry | 7.47E-07-1.8E-04 |
| Hepatic System Disease | 8.77E-12-9.65E-05 | Tumor Morphology | 3.38E-06-1.1E-05 |
| Metabolic Disease | 9.01E-11-9.72E-05 | Cell-mediated Immune Response | 8.41E-06-2.56E-05 |
| Reproductive System Disease | 2.97E-10-1.85E-04 | Hair and Skin Development and Function | 1.5E-05-1.5E-05 |
| Immunological Disease | 3.19E-10-1.8E-04 | Renal and Urological System Development and Function | 1.59E-05-1.59E-05 |
| Inflammatory Disease | 3.19E-10-1.8E-04 | DNA Replication, Recombination, and Repair | 2.8E-05-4.8E-05 |
| Respiratory Disease | 3.19E-10-1.44E-04 | Embryonic Development | 6.85E-05-1.38E-04 |
| Renal and Urological Disease | 6.43E-10-1.84E-04 | Organismal Development | 6.85E-05-1.38E-04 |
| Inflammatory Response | 2.17E-09-1.8E-04 | Post-Translational Modification | 1E-04-1E-04 |
| Connective Tissue Disorders | 5.98E-09-1.44E-04 | Carbohydrate Metabolism | 1.1E-04-1.23E-04 |
| Infectious Diseases | 1.69E-08-1.39E-04 | Lipid Metabolism | 1.1E-04-1.23E-04 |
| Ophthalmic Disease | 1.91E-08-1.8E-04 | Hypersensitivity Response | 1.6E-04-1.6E-04 |
| Behavior | 3.21E-08-1.04E-05 | Cellular Assembly and Organization | 1.88E-04-1.88E-04 |
| Molecular Transport | 6.37E-08-1E-04 | Cellular Function and Maintenance | 1.88E-04-1.88E-04 |
| Cell-To-Cell Signaling and Interaction | 6.47E-08-6.8E-05 | | |

**Table S5 The different expressed mRNAs enriched into the sphingolipid metabolism (APP/PS1).**

| Ingenuity Canonical Pathways | -log(p-value) | Molecules |
|---|---|---|
| 1D-myo-inositol Hexakisphosphate Biosynthesis II (Mammalian) | 1.76 | INPP5J, INPP5D, ITPKB, ITPKA, INPP5A |
| 3-phosphoinositide Biosynthesis | 0.901 | DUSP10, PTPRM, PIK3R3, PHOSPHO1, CDC25A, IRS1, PTPN22, KIT, VAV1, EPHX2, CD28, DUSP5, DUSP14, PPP1R1A, PPP1R1B, CD86, PIP5K1B, SGPP2, PXYLP1, PTPN7, DUSP16 |
| 3-phosphoinositide Degradation | 1.11 | DUSP10, INPP5J, PTPRM, PHOSPHO1, CDC25A, PTPN22, EPHX2, MTM1, INPP5D, DUSP5, DUSP14, PPP1R1A, INPP4B, PPP1R1B, SGPP2, PXYLP1, PTPN7, DUSP16 |
| Adipogenesis pathway | 1.08 | AGPAT2, KAT2B, FGF1, FZD2, FZD7, SMAD9, KAT6A, LPIN1, SLC2A4, FZD1, CTBP2, SAP30, EBF1, LPL, RPS6KA1, KLF5 |
| Ceramide Biosynthesis | 0.341 | SPTSSB |
| Ceramide Degradation | 0.341 | ASAH2 |
| Ceramide Signaling | 0.287 | TNFRSF11B, FOS, PIK3R3, SMPD3, CERK, MRAS, MAP3K1, IRS1 |
| Complement System | 1.13 | C1QC, ITGAM, CFB, C3, C1QB, ITGAX |
| CREB Signaling in Neurons | 3.63 | GNG11, PRKAR2B, MRAS, GNG10, GNG7, IRS1, PLCE1, GRIN2C, GRM3, GRM4, GNG3, PRKCB, ADCY2, ITPR1, GRM1, GNG13, PRKCH, GRIA4, GRM5, NOTUM, GRIK5, PIK3R3, ADCY5, PLCB4, CAMK4, CAMK2A, ITPR3, GRID2, RPS6KA1, PLCZ1 |
| GDNF Family Ligand-Receptor Interactions | 1.64 | FOS, DOK4, PIK3R3, ITPR3, GFRA1, MRAS, MAPK12, RET, GFRA4, DOK6, IRS1, ITPR1 |
| GDP-glucose Biosynthesis | 0.3 | HK2 |
| Glucose and Glucose-1-phosphate Degradation | 0.265 | HK2 |
| Glutathione-mediated Detoxification | 0.218 | GSTT2/GSTT2B, HPGDS |
| Glycoaminoglycan-protein Linkage Region Biosynthesis | 0.341 | B3GAT1 |
| Granulocyte Adhesion and Diapedesis | 1.03 | ITGAM, ITGAL, MMP9, ITGA3, EZR, CLDN19, MMP17, CCL21, CCL27, CX3CL1, SDC1, TNFRSF11B, IL18, HRH3, SELPLG, IL1RL2, CXCL14, MMP23B, MMP24 |

| | | |
|---|---|---|
| NAD Phosphorylation and Dephosphorylation | 0.236 | PXYLP1 |
| Phenylalanine Degradation I (Aerobic) | 0.532 | PCBD1 |
| Phospholipase C Signaling | 1.48 | GNG11, ITGA3, RHOD, PLD3, MRAS, GNG10, GNG7, PLCE1, LCP2, SYK, GNG3, PRKCB, ADCY2, ITPR1, GNG13, PRKCH, BLNK, FNBP1, MYL4, ADCY5, PLCB4, CAMK4, PLA2G3, MEF2C, FCGR2A, ITPR3, PPP3R1, CD247 |
| Phospholipases | 1.27 | PNPLA3, PLCB4, PLD3, PLCE1, PLCZ1, PLB1, PLA2G3, NOTUM, LIPG |
| Renin-Angiotensin Signaling | 2.36 | PIK3R3, ADCY5, PRKAR2B, MRAS, MAPK12, IRS1, AGTR2, SHC3, AGT, FOS, ITPR3, PAK6, PTK2B, MAP3K1, MAPK13, PRKCB, ADCY2, ITPR1, PRKCH |
| Sphingomyelin Metabolism | 0.3 | SMPD3 |
| Sphingosine and Sphingosine-1-phosphate Metabolism | 0.772 | ASAH2, SGPP2 |
| Sphingosine-1-phosphate Signaling | 0.861 | PIK3R3, SMPD3, ADCY5, RHOD, PLCB4, ASAH2, IRS1, PLCE1, PDGFB, PTK2B, ADCY2, PLCZ1, NOTUM, FNBP1 |
